# Mucosal Tolerance to a Combination of ApoB and HSP60 Peptides Controls Plaque Progression and Stabilizes Vulnerable Plaque in Apob^tm2Sgy^Ldlr^tm1Her^/J Mice

**DOI:** 10.1371/journal.pone.0058364

**Published:** 2013-03-11

**Authors:** Lakshmi Mundkur, Rupak Mukhopadhyay, Sonia Samson, Meenakshi Varma, Dnyaneswar Kale, Daxin Chen, Sneha Shivaprasad, Hemapriya Sivanandan, Vinod Soman, Xinjie Lu, Vijay V. Kakkar

**Affiliations:** 1 Mary and Gary Western and Tata Molecular Immunology Unit, Thrombosis Research Institute, Bangalore, India; 2 Molecular Immunology Unit, Thrombosis Research Institute, London, United Kingdom; Universität Würzburg, Germany

## Abstract

Oral tolerance to auto antigens reduces the development of atherosclerosis in mouse models. However, the effect of immune tolerance to multiple self antigenic peptides in plaque progression and stabilization is not known. We studied the protective effect of mucosal tolerance to peptides from apolipoprotein B (ApoB; 661–680) and heat shock protein 60 (HSP60; 153–163), in combination with diet, in the prevention of atherosclerotic lesion progression and plaque stabilization in ApoB^tm25gy^LDLr^tm1Her^ mice. We found that oral administration of five doses of a combination of ApoB and HSP60 peptides (20 µg/mice/dose) induced tolerance to both the peptides and reduced early plaque development by 39.9% better than the individual peptides (ApoB = 28.7%;HSP60 = 26.8%)(P<0.001). Oral tolerance to combination of peptides along with diet modification arrested plaque progression by 37.6% which was associated with increases in T-regulatory cell and transforming growth factor-β expression in the plaque and peripheral circulation. Reduced macrophage infiltration and tumor necrosis factor-α expression in the plaque was also observed. Tolerance with continued hypercholesterolemia resulted in 60.8% reduction in necrotic core area suggesting plaque stabilization, which was supported by reduction in apoptosis and increased efferocytosis demonstrated by greater expression of receptor tyrosine kinase Mer (MerTK) in the plaque. Tolerance to the two peptides also reduced the expression of matrix metalloproteinase 9, tissue factor, calprotectin, and increased its collagen content. Our study suggests that oral tolerance to ApoB and HSP60 peptide combination induces CD4^+^ CTLA4^+^ Tregs and CD4^+^CD25^+^Foxp3^+^ Tregs secreting TGF-β, which inhibit pathogenic T cell response to both peptides thus reducing the development and progression of atherosclerosis and provides evidence for plaque stabilization in ApoB^tm25gy^LDLr^tm1Her^ mice.

## Introduction

Atherosclerosis is a chronic inflammatory disease of the arterial wall characterized by accumulation of lipids and immune inflammatory cells [1], [2], [3]. Inflammation mediated by a pathogenic T-cell response to autologous antigens like modified low density lipoproteins (LDL) and heat shock proteins (HSP) as well as exogenous antigens from pathogens, have been implicated in the initiation of an autoimmune response during atherogenesis [4], [5], [6], [7], [8]. HSP60 is a highly conserved protein expressed by cells in response to stress. Recently it was shown that HSP60-reactive T-cells can initiate atherosclerosis by recognizing atherogenic HSP60 epitopes in the intima [9]. Retention of LDL in the arteries and its modification are early events during atherogenesis which expose neo epitopes from ApoB 100 and Oxidized LDL, initiating inflammatory adaptive T cell response [7], [10]. Pro inflammatory Th1 response to these antigens thus predominates during the progression of the disease.

The immune system generates regulatory T cells (Tregs), which actively suppresses immune activation and maintains immune homeostasis [11], [12]. Imbalance in the pathogenic T cells producing proatherogenic mediators and Tregs with immunosuppressive properties is well established during the development of disease [2], [13], [14]. It is now established that loss of immunological tolerance against antigens in the plaque results in proinflammatory autoimmune response [15], [16]. Immunotherapy for atherosclerosis is directed toward inducing tolerance to self-antigens mediated by protective antibodies or increasing the number of antigen-specific Treg cells, which can suppress the proatherogenic T-effector cells [16], [17], [18], [19].

Mucosal route of administration is an attractive method of inducing antigen-specific peripheral tolerance to treat autoimmune diseases [20]. The effects of tolerance are measured as reduction in systemic T cell response, secretion of the anti-inflammatory cytokines interleukin-10 (IL-10) and transforming growth factor-β (TGF-β), and suppression of antibody response [21]. Several studies have demonstrated effective early reduction of atherosclerosis in hyperlipidemic mouse models by inducing tolerance to modified lipids and peptides derived from apolipoprotein B (ApoB) 100, HSPs 60/65, and β2-glycoprotein [22], [23], [24], [25], [26], [27], [28]. However, the effect of immune tolerance on plaque regression and stabilization has not been studied in detail.

Vulnerable plaques are characterized by a thin fibrous cap, higher expression of markers such as calprotectin, (Mrp8/14) [29], matrix metalloproteinases (MMPs),extensive calcification [30], inflammatory cytokines, and apoptosis of intimal cells, leading to an expansion of a lipid-laden necrotic core [31]. Endoplasmic reticulum stress-induced apoptotic cell death in advanced atherosclerotic plaques, coupled with defective clearance (efferocytosis) resulting in the release of proinflammatory and prothrombotic markers, has been reported in several studies [32], [33].

We have earlier reported that repeated subcutaneous immunization with a combination of peptide epitopes from ApoB100 and HSP60 could significantly reduce early atherosclerosis development in mice compared to individual peptides [34]. We chose the ApoB peptide based on previous observations that immunization with this peptide (AA 661-680) was atheroprotective [35]. The human HSP60 epitope was chosen based on its sequence similarity with mouse HSP60 and high degree of homology with those from other eukaryotes and prokaryotes. We believe that a multifaceted disease like atherosclerosis should be addressed with multiple antigenic epitopes to have maximum efficacy.

In the present study we show that oral tolerance to a combination of ApoB and HSP60 peptides induces an antigen-specific Treg response, arrests progression of the established plaque, and stabilizes the mature plaque as evidenced by reduction in the necrotic area, apoptosis, and reduction in the expression of plaque vulnerability markers in a double-gene knockout (ApoB^tm25gy^/LDLr^tm1Her^) mouse model. This model was chosen as it expresses only ApoB-100 and is deficient in the LDL receptor, with most of the cholesterol being transported in apoB100-containing lipoproteins, and it is reported to more closely mimic human atherosclerosis than other models [36].

## Methods

### Animals

This study was carried out in strict accordance with the recommendations in the Guide for the Care and Use of Laboratory Animals of the Committee for the Purpose of Control and Supervision of Experiments on Animals (CPCSEA), Ministry of Environment, Government of India and conforms to the Guide for the Care and Use of Laboratory Animals published by the US National Institutes of Health (NIH Publication, 8th Edition, 2011). The protocol was approved by the Institutional Animal Ethics Committee of the Thrombosis Research Institute (Registration Number: 1261/c/09/CPCSEA).

ApoB^tm2Sgy^/Ldlr^tm1Her/J^ knockout mice on a C57BL/6 background (Jackson labs) were kept under standard laboratory conditions and fed a normal chow diet (Nutrilab, India) or a high-fat diet (Harlan, TD 96121 Indianapolis, USA). Food and water were administered *ad libitum*. Tolerance induction was carried out twice in groups of 6–8 mice/group per experiment.

### Antigens and Experimental Design

**Figure 1:**
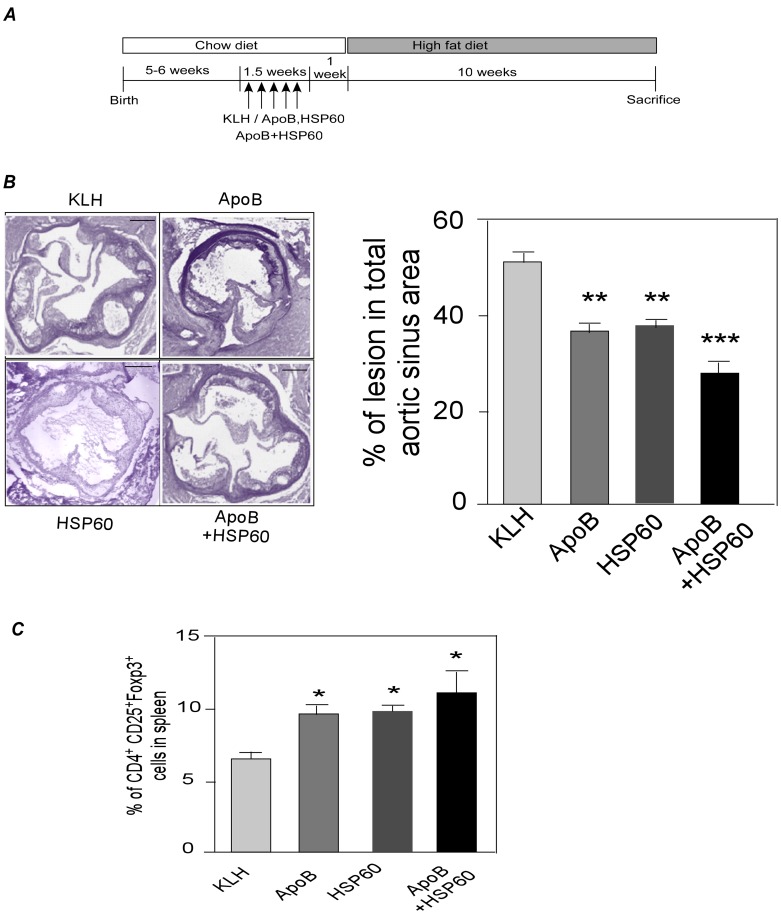
Oral Administration of Combination of ApoB and HSP60 Peptides Provides Improved Efficacy against Atherosclerosis Compared to Individual Peptides. A. Experimental design. B. Representative photomicrographs of EVG stained plaque area and its quantitative analysis in aortic sinus of 18 week old ApoB^tm25gy^LDLr^tm1Her^ mice (n = 10 per group). Scale bar represents 200 µm. C. Percentage of CD25^+^Foxp3^+^ cells (*P<0.05) within the CD4 population in spleen at the end of the study using flow cytometry analysis (n = 6 per group).

**Figure 2:**
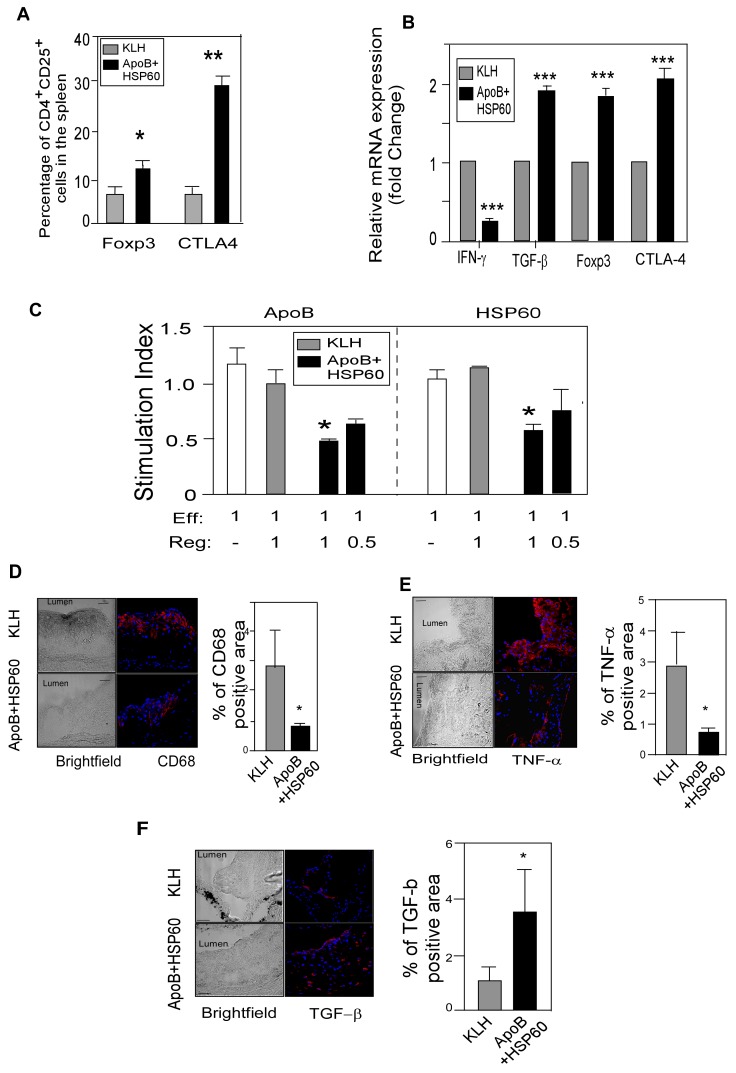
Oral Administration of ApoB+HSP60 Peptides Induces Tolerance to Both Peptides and attenuates inflammation. A. Flow cytometry analysis: Percentage of CD25^+^Foxp3^+^ cells (*P = 0.035) and CTLA-4^+^cells (**P<0.0001) within the CD4 population in spleen at the end of the study (n = 6 per group). B. Relative mRNA expression of IFN-γ (*** P<0.001), TGF-β (*** P<0.005), Foxp3 (*** P<0.005), and CTLA-4(*** P<0.005) in the ascending aorta quantified by RT-PCR analysis and normalized to GAPDH. Fold-changes in their expression in ApoB+HSP60-tolerized mice relative to controls are shown (n = 5 per group). C. Splenic effector cells were generated from ApoB/Ldlr^−/−^ mice immunized subcutaneously with the peptides. Addition of purified Treg cells from oral tolerant mice is indicated at different ratios to effector cells. Proliferation of effector-cell alone is indicated by the white bar; proliferation index represents the percentage carboxyfluorescein succinimidyl ester reduction in culture stimulated with ApoB or HSP60 peptides (10 µg/mL) relative to unstimulated culture (n = 4 per group). *P<0.05 D. Representative photomicrographs showing immunofluorescence staining of aortic sinus sections with CD68 (red) and its quantitative analysis (n = 10 per group). *P = 0.02. E. Representative photomicrographs showing immunofluorescence staining of aortic sinus sections with TNF-α (red) and its quantitative analysis (n = 6 per group). *P = 0.04. F. Representative photomicrographs showing immunofluorescence staining of aortic sinus sections with TGF-β (red) and its quantitative analysis (n = 6 per group). *P = 0.03. Scale bar represents 50 µm for the immunofluorescence staining.

The antigens used were apolipoprotein B -keyhole limpet hemocyanin (ApoB -KLH) peptide (ApoB-100 amino acids 661–680 conjugated to KLH, used at 1 mg/mL, dissolved in phosphate-buffered saline (PBS) and heat shock protein 60-KLH (HSP60-KLH) peptide (HSP60 amino acids 153–163 conjugated to KLH, used at 1 mg/mL, dissolved in PBS) (Severn Biotech, Worcester, UK). The two peptides were combined in equal concentrations (10 µg each) and 20 µg of the combination was administered orally, per animal, per dose. KLH (Sigma chemicals, St. Louis, USA) was used as the control, at 20 µg per animal, per dose. Five- to 6-week-old ApoB^tm2Sgy^/Ldlr^tm1Her^ J mice in a C57Bl6/J background were given five doses of a combination of peptides or KLH. Mice were fed either a chow diet or a diet high in fat and cholesterol (Harlan, TD 96121 Indianapolis, USA (21% fat and 1.25% cholesterol), as described in the figures 1A, 2A and 4A.

### Lipid Analysis

Blood was collected by retro-orbital venous plexus under 3% Isoflurane inhalant anesthesia in oxygen as per American Veterinary Medical Association guidelines (June 2007). Lipid concentrations of plasma total were determined using the Cobas Fara II Clinical Chemistry auto analyzer (F. Hoffman La Roche Ltd, Basel, Switzerland), following the manufacturer’s instructions.

### Atherosclerotic Lesion Assessment

Quantification of atherosclerotic lesions was carried out as per the protocol approved by the Animal Models of Diabetic Complications Consortium (http://www.diacomp.org). Mice were euthanized humanely using an overdose of isoflurane inhalant anesthetic (15%) as per American Veterinary Medical Association guidelines (June 2007). Mouse hearts were perfused with 10 mL of PBS and collected in either optimal cutting temperature (OCT) medium (Tissue Tek, Leica, Germany) and neutral buffered phenol (NBF). Aortic root sections (10 µm) were cut from the hearts embedded in an OCT medium in frozen conditions using a cryotome (Leica CM 1900 UV Cryotome) and from hearts in neutral buffered phenol (NBF) after embedding in paraffin blocks. For lesion analysis in each mouse, five sections 80 µm apart were stained with Elastica van Geison (EVG). Total area and area covered with Lesion were calculated using Image-Pro Plus software (Media Cybernetics, Bethesda, MD). Plaque necrosis was quantified by measuring the size of the hematoxylin and eosin-negative acellular area, as described previously [37]. Whole aortas were collected in NBF and used for enface analysis using Oil red-O staining as described previously [38]. Briefly, the aortas were removed, cut open, fixed in 10% buffered formalin, and stained with Oil red-O. The en-face lesion area of the aorta was quantified relative to its surface area using Image-Pro Plus software. Lesion area was expressed as a percentage of total plaque area. Three aortas from each group of mice were collected in RNA later (Ambion, Austin, TX, USA) and snap frozen for RNA extraction.

### Immunohistochemical Analysis of Atherosclerotic Lesions

Immunofluorescence on frozen sections was carried out using an indirect immunofluorescence technique. The frozen sections on poly-l-lysine-coated slides (poly-prep-slides Sigma) were permeabilized using 0.2% of triton X 100 for 30 min, fixed with ice-cold acetone, and blocked with 5% goat serum or 5% rabbit serum depending on the secondary antibody used. Further, the sections were incubated with primary antibodies (Rat Anti-mouse CD68 (AbD Serotech, Oxford, UK) Mouse monoclonal to TNF-α (Abcam, Cambridge, UK), Rabbit Polyclonal to TGF-beta Abcam, Cambridge, UK) Mouse monoclonal to macrophage L1 protein calprotectin (Abcam, Cambridge, UK) for 2 h followed by incubation with an appropriate secondary antibody (Alexa-633 (Invitrogen, Carlsbad, California, USA) for 1 h. Sections were mounted with Vector Shield. Images were captured using a Leica DMI 4000 B confocal microscope and the analysis was done using Image-Pro software, and percentage areas of fluorescence of specific antigens of interest in the plaque were calculated.

Frozen sections of aortic root were fixed and stained using double indirect immunofluorescence to measure the CD4^+^ Foxp3^+^ cells in the plaque. For Foxp3+-CD4 T-cells FITC-conjugated rat anti-mouse CD4 mAb (BD Biosciences, New Jersey, USA) and phycoerythrin (PE)-labeled anti-mouse Foxp3 (BioLegend, San Diego, CA, USA) were used. All slides were counter-stained with mounting medium containing 4, 6-diamino-2-phenylindole (DAPI) (Vector Laboratories Inc., Peterborough, UK). When counting the numbers of cells in atherosclerotic lesions, the 10 to 12 largest lesions present in aortic root were selected for analysis. Cell numbers were counted, calculated and expressed as the Mean ± SEM. CD4 and Foxp3 staining was performed as described previously [34].

### Alizarin Red Staining for Calcium Content

The amount of calcium deposited in the necrotic core of the aortic sinus was evaluated with Alizarin Red S (Sigma chemicals, St. Louis, USA). Paraffin sections of the aortic root were deparaffinized and stained with Alizarin Red S for 3 min followed by acetone and xylene treatment and mounted in DPX (mixture of distyrene, tricresyl phosphate, and xylene). The percentage of calcified area (stained red) was measured by the ratio of the area of calcium deposition (stained red) to the total plaque area using Leica DMI 4000 B confocal microscope. Captured images were analyzed using Image-Pro Plus software (Media Cybernetics).

### Masson’s Trichrome Staining for Collagen Content

Tissue sections were treated with Bouin’s solution to intensify the final color. Nuclei were stained with Weigert’s iron hematoxylin, and cytoplasm and muscle were then stained with Beibrich Scarlet-Acid Fuchsin after treatment with phosphotungstic/phosphomolybdic acid. The presence of collagen was demonstrated by staining with aniline blue.

### Plasma Cytokine Concentrations

Cytokine concentrations in the plasma of individual mice such as IL-10, TGF-β, IFN-γ, and TNF-α were measured using paired antibodies specific for the corresponding cytokines by ELISA kits, as per the manufacturer’s instructions (eBiosciences, CA, USA).

### Apoptosis Assay

Caspase-3 staining was performed using an immunohistochemical staining protocol on paraffin sections of the aortic sinus by Caspase-3 mouse monoclonal antibody reacting to both pro-Caspase 3 and activated Caspase 3 larger subunit (Imgenex San Diego, California, USA) as described in the immunostaining section. Double-staining for αSMA (Abcam, Cambridge, UK) and caspase-3 or F4/80 (Abcam, Cambridge, UK) and caspase-3 were carried out using double indirect immunofluorescence. When counting the numbers of cells in atherosclerotic lesions, the 10 to 12 largest lesions present in aortic root were selected for analysis. Cell numbers were counted, calculated and expressed as the Mean ± SEM.

### Flow Cytometry

For fluorescent activated cell scanner (FACS) analyses, splenocytes were isolated from control and peptide-treated mice at the end of the study. To study oral tolerance, splenocytes were isolated 1 week after the final dose. Cells were stained in 2% serum containing phosphate buffered saline. Flow cytometry analyses were performed by FACS Canto II using FACS DIVA software (Becton Dickinson, New Jersey, USA) and FLOWJO software (Tree star Ltd, Oregon, USA). The antibodies used were as follows: Fluorescein isothiocyanate (FITC)-conjugated CD4 (clone RM4-5; eBiosciences, San Diego, CA, USA), allophycocyanin (APC)-anti CD25 (clone PC61.5; eBiosciences), phycoerythrin (PE)-anti-forkhead box p3 (Foxp3) (clone NRRF-30, eBiosciences), allophycocyanin(APC)-anti-TGF-β1 (R&D systems, Minneapolis, MN, USA) PE-anti-CD152 (clone UC10-4F10-11), and isotype-matched control antibodies. Intracellular staining of Foxp3 was performed using the Foxp3-staining buffer set (eBiosciences) according to the manufacturer’s instructions. Surface staining was performed according to standard procedures at a density of 1×10^5^ cells/100 uL, and volumes scaled up accordingly.

### Cell Proliferation and Cytokine Assays

Cell culture experiments were performed in Rosewell Park Memorial Institute (RPMI) 1640 medium (Bio Whittaker, Walkersville, MD, USA) supplemented with 10% Fetal bovine serum, 2 mM glutamine, 10 mM HEPES (4-(2-hydroxyethyl)-1-piperazineethanesulfonic acid), sodium pyruvate, and antibiotics. X vivo 20 (Lonza, Basel, Switzerland) was used for the assays whenever the supernatants were collected for cytokine analysis. Splenocytes and lymph node cells were passed through a sterile cell strainer, washed twice with Hanks balanced salt solution and plated in culture dishes at a concentration of 1×10^5^ cells/mL in RPMI medium and stimulated with 10 µg/mL of concavalin A (ConA; Merck, New Jersey, USA). Culture supernatants were collected at 72 h, acidified by the addition of HCl, and neutralized with NaOH. Transforming growth factor (TGF)-β concentrations were measured by enzyme-linked immunosorbent assay (ELISA) kits, as per the manufacturer’s instructions (eBiosciences, California USA). The concentrations of interferon (IFN)-γ and interleukin (IL) 10 were also measured in the supernatant by ELISA as per the manufacturer’s instructions (eBiosciences, California, USA).

### Functional Immunoassays

To generate effector T-cells, groups of six mice were immunized with either ApoB–KLH peptide or HSP60–KLH peptide (50 µg/animal) via the subcutaneous route with complete Freund’s adjuvant. The animals were given two booster doses of the same antigen (25 µg/animal) in incomplete Freund’s adjuvant 3 weeks apart. Six days after the last immunization, the splenocytes were collected and used as effector cells. Oral tolerance was induced in a second set of mice as described earlier. The spleen cells were collected from tolerized mice and regulatory T cells were isolated using a CD4+CD25+ regulatory T Cell Isolation Kit (Miltenyi Biotech, Teterow, Germany). The CD4+CD25+ regulatory cells were labeled with 6 µM PKH26 (Sigma chemicals, St. Louis, USA) to discriminate the effector and regulatory CD4 population. The effector cells were labeled with 10 µM CFSE (Sigma chemicals). The effector cells (1×10^5^) and regulatory cells were taken in different ratios, and activated with 10 µg/mL of antigen (ApoB100 peptide and HSP60 peptide). After 5 days of incubation, cells were stained with CD4-APC (eBiosciences, California, USA) [39].

The lymphocytes were gated using forward and side-scatter plots. PKH26 (Sigma, USA)-stained CD4 cells were excluded from the analysis. Proliferation of CD4 effector cells was measured by 5,6-carboxyfluorescein diacetate succinimidyl ester (CFSE) dilution using FACS CANTO II (Becton Dickinson, New Jersey, USA) and analyzed using FLOWJO software. The proliferation index of T cells was calculated as described previously [40].

### Real-time Reverse Transcription Polymerase Chain Reaction (RT-PCR) Analysis

Total RNA was extracted from the ascending part of the aorta. RNA was extracted using TRIzol reagent (Invitrogen, Carlsbad, California, USA). RT-PCR was performed with two-step EXPRESS SYBR superscript RT-PCR kit (Invitrogen, Carlsbad, California, USA) using the ABI PRISM 7500 sequence detection system (Applied Biosystems, 7500 real time PCR system) according to the manufacturers protocol using the standard cycling program. Amplification reactions were performed in triplicates from RNA isolated from three mice per experimental group and the fluorescent curves were analyzed with the included software. The sequences of primers used are given in Methods S3.

### Statistical Analysis

Data are expressed as Mean ± SEM. Differences between control and treated groups were evaluated by Mann- Whitney test and were and are considered statistically significant at P<0.05. Statistical analyses were performed using Graph Pad prism software version 5.01(GraphPad Software, Inc., La Jolla, CA, USA).

## Results

### Oral Administration of Combination of ApoB and HSP60 Peptides Provides Improved Efficacy against Atherosclerosis Compared to Individual Peptides

We first studied the effect of oral administration of individual peptides derived from ApoB and HSP60 and their combination (ApoB+HSP60) on development of atherosclerosis in ApoB^tm2Sgy^/Ldlr^tm1Her^ mice (Figure 1A). Individual treatment with ApoB and HSP60 peptides reduced early lesion development by 28.7%, and 26.8%, respectively, while the combination of two peptides resulted in a significant reduction in atherosclerotic lesion in aortic root (39.9%, P<0.001) (Figure 1B). This reduction was independent of circulating lipid levels (Table S1A). Oral administration of individual peptides and their combination was found to increase the Treg cell population (P<0.01) in the spleen of treated animals as observed by flow cytometry (Figure 1C). As the lesion area in KLH-treated mice was comparable to PBS, KLH was taken as the control in all experiments (Figure S1B). Since the combination of peptides was more effective in reducing early atherosclerosis development we used only the peptide combination for subsequent experiments.

### Oral Administration of ApoB+HSP60 Peptides Induces Tolerance to Both Peptides and Attenuates Inflammation

We next examined the effect of oral dosing on with peptide combination on tolerance induction to both peptides. Analysis of Treg cell population by flow cytometry showed 41.2% (P<0.001) increase in the number of CD4+CD25+Foxp3+ T cells and CTLA4+ T cells in the spleen, mesenteric lymph nodes and peripheral blood cells, in peptide-tolerated mice compared to control (Figures 2A, S2A,S2B and S2C), suggesting immunological tolerance to peptides. Consistent with these findings we also observed an increase in the expression of TGF-β, Foxp3, and CTLA-4 mRNA by 1.9-fold, 1.8-fold, and 2.0-fold, respectively (P<0.005 for each) in the aorta of peptide tolerated mice compared with KLH (Figure 2B), suggesting an increase in number of Treg cells in the plaque. We assessed the ability of Treg cells recovered from either KLH or ApoB+HSP60-tolerized mice to suppress the proliferation of T effector CD4^−^CD25^−^ cells recovered from peptide sensitized mice. A dose-dependent reduction in effector T-cell proliferation was observed after co culture with Treg cells recovered from peptide-tolerated animals in the presence of both ApoB peptide and HSP60 peptide (P<0.05 for both) but no reduction was observed with Treg cells from controls (Figure 2C), suggesting an expansion of antigen specific Treg cells in peptide tolerated mice. Antibodies specific for ApoB and HSP60 peptides were not detected in the serum after oral administration of peptides, further supporting the induction of tolerance to individual peptides (Table S2). Splenocytes stimulated with mitogen (concavalin A) showed an increase in TGF-β concentration in the supernatants of peptide-tolerated mice compared with controls (P = 0.017), while IL-10 concentrations did not differ between the two groups (Figure S2D).

We next examined the cytokines and macrophage infiltration in the lesion by immunohistochemical analysis of aortic sinus. The percentage of CD68-postive and TNF-α-positive areas was reduced significantly, by 77% (P = 0.02) and 64% (P = 0.04), respectively (Figure 2D and 2E), while an 80% increase (P = 0.03) in the TGF-β–positive area was observed in the aortic sinus of peptide-tolerated mice compared with controls (Figure 2F). Plasma concentrations of IFN-γ were lower (P = 0.02) while that of TGF-β were significantly higher in the peptide-tolerated mice (P = 0.01) compared to KLH (Figure S2E, and S2F).

### Oral Tolerance to ApoB+HSP60 Peptides in Combination with Diet Modification Arrests Plaque Progression

**Figure 3:**
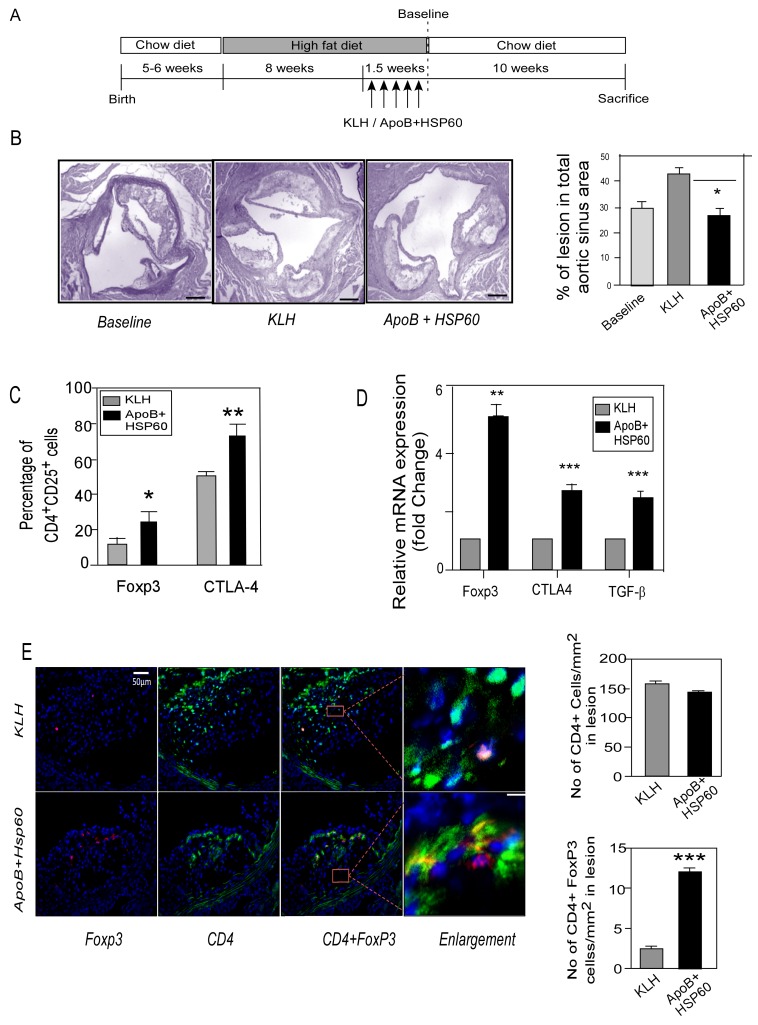
Oral Tolerance to ApoB+HSP60 peptides in Combination with Diet Modification Arrests Plaque Progression. A. Experimental design. B. Representative photomicrographs of EVG stained plaque area and its quantitative analysis in aortic sinus of 26 week old ApoB^tm25gy^LDLr^tm1Her^ mice (n = 10 per group) *P = 0.01 for APOB+HSP60 tolerized animals compared to KLH. Scale bar represents 200 µm. C. Percentage of CD25^+^Foxp3^+^ cells (*P = 0.011) and CTLA-4^+^cells (**P = 0.005) within the CD4 population in spleen (n = 6 per group). D. Expression of mRNA of Foxp3 (**P = 0.003), CTLA-4 (***P<0.001), and TGF-β (***P<0.002) in the ascending aorta were quantified by RT-PCR analysis and normalized to GAPDH. Fold-changes in their expression in ApoB+HSP60-tolerized mice relative to controls (n = 5 per group). E. Representative photomicrographs showing double immunofluorescence staining of aortic sinus sections with CD4 (green) and Foxp3 (red). Scale bar represents 50 µm. Enlarged region to show double immune staining. Scale bar represents 6.25 µm. Lower panel: Number of CD4-positive cells/mm^2^ and CD4+ Foxp3+ cells/mm^2^ (n = 9 per group), ***P<0.001.

To study the effect of oral tolerance to the peptides on plaque progression, ApoB^tm2Sgy^/Ldlr^tm1Her^^−^ mice were fed a high-fat diet for 10 weeks, to establish atherosclerotic lesion. In the last 1.5 weeks, mice were orally dosed with peptides or KLH followed by a shift to chow diet for the next 10 weeks (Figure 3A). At baseline (before shifting the mice to chow diet), the mice had a plasma cholesterol concentration of 14.10±0.52 mMol/L and had established atherosclerotic lesions in the aortic sinus (29.48±3.6%) (Figure 3B). Total plasma cholesterol reduced in both control and treated groups on shifting to chow diet (10.79±2.65 mMol/L in controls and to 9.91±0.15 mMol/L with peptide treatment) (Table S1B). Despite diet control, the lesion continued to increase in KLH (42.93±2.9%) and PBS control mice (38.49±2.62%) (Figure S3A). Tolerance to peptides resulted in a 37.6% reduction in lesion progression compared with KLH in the aortic root (42.93±2.9% vs. 26.8±4.4%, P = 0.01) (Figure 3B) and descending aorta (Figure S3B).

Flow cytometry analysis of revealed an increase in CD4^+^ CD25^+^ T cells expressing Foxp3 (P = 0.011) and CTLA-4 (P = 0.005) in the spleens of tolerized animals (Figure 3C and S4A). Consistent with these observations, the expression of regulatory cell markers;Foxp3 CTLA4 and TGF-β were significantly higher in aorta as shown by RT PCR analysis(Figure 3D and the number of CD4^+^ Foxp3^+^ regulatory T cells was higher (2.53±0.14 vs. 12.09±0.43, P<0.001) in the aortic sinus of the peptide-tolerized mice as observed by immunohistochemistry (Figure 3E).

### Shift in the Balance between Pro- and Anti-Inflammatory Markers in Association with Plaque Regression

**Figure 4:**
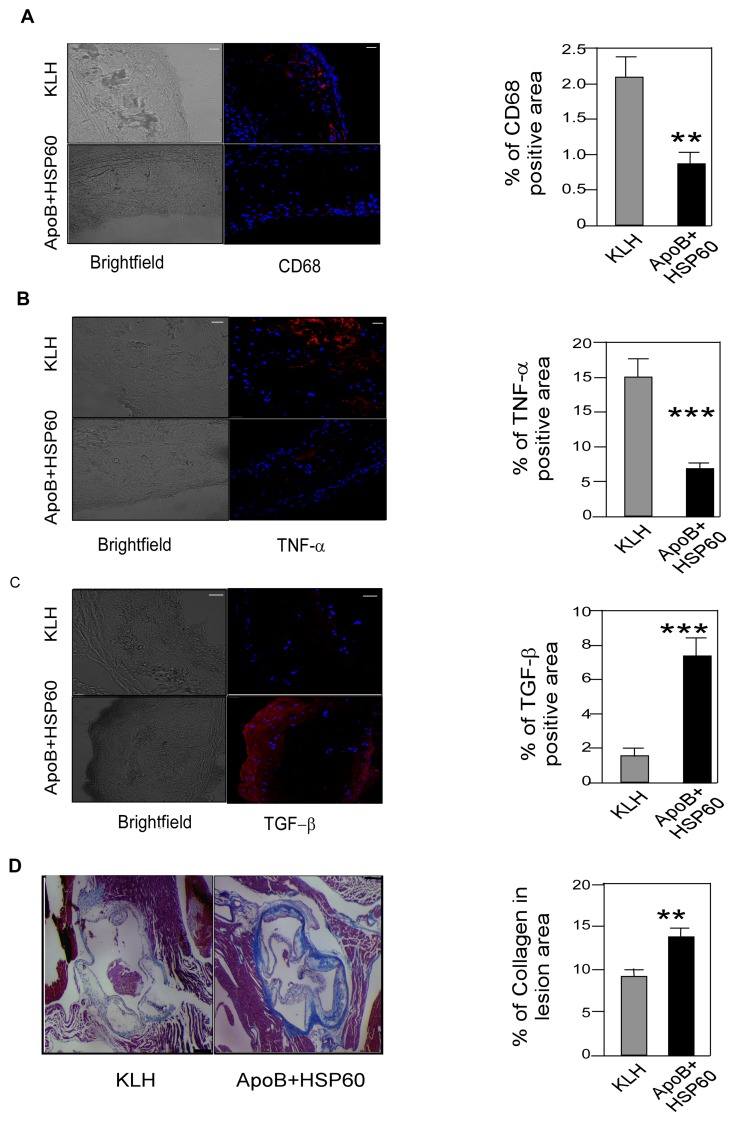
Shift in the Balance between Pro- and Anti-Inflammatory Markers in Association with Plaque Regression. A. Representative photomicrographs showing immunofluorescence staining of aortic sinus sections with CD68 (red) and its quantitative analysis (n = 10 per group). **P = 0.025 B. Representative photomicrographs showing immunofluorescence staining of aortic sinus sections with TNF-α (red) and its quantitative analysis (n = 6 per group). ***P = 0.001 C. Representative photomicrographs showing immunofluorescence staining of aortic sinus sections with TGF-β (red) and its quantitative analysis (n = 6 per group). ***P = 0.003 D. Paraffin-embedded aortic sinus sections stained with Masson’s trichrome (light blue) and quantitative analysis of collagen in lesion area (n = 6 per group). **P = 0.008. Scale bar represents 200 µm. Scale bar represents 50 µm for the immunofluorescence staining.

To confirm that the arrest in plaque progression is actually due to reduced inflammation in the plaque, we studied macrophage infiltration and cytokine expression in the aortic sinus. The percentage of macrophage CD68 and TNF-α–positive areas was reduced significantly, by 58.8% (P = 0.025) and 67.12% (P = 0.001), respectively, following oral tolerance (Figure 4A, 4B). The reduction of inflammation was also supported by a 79.8% increase (P = 0.003) in the TGF-β–positive area in the aortic sinus of peptide-tolerized mice compared with controls (Figure 4C). Plasma concentration of TGF-β (P = 0.02) was higher in tolerized- mice compared to controls (Figure S4B). Tolerance to peptides also resulted in 33.3% increase in collagen content in the lesion (P = 0.008), suggesting plaque stability (Figure 4D).

### Oral Tolerance to ApoB+HSP60 Peptides Stabilizes Advanced Atherosclerotic Lesion

**Figure 5:**
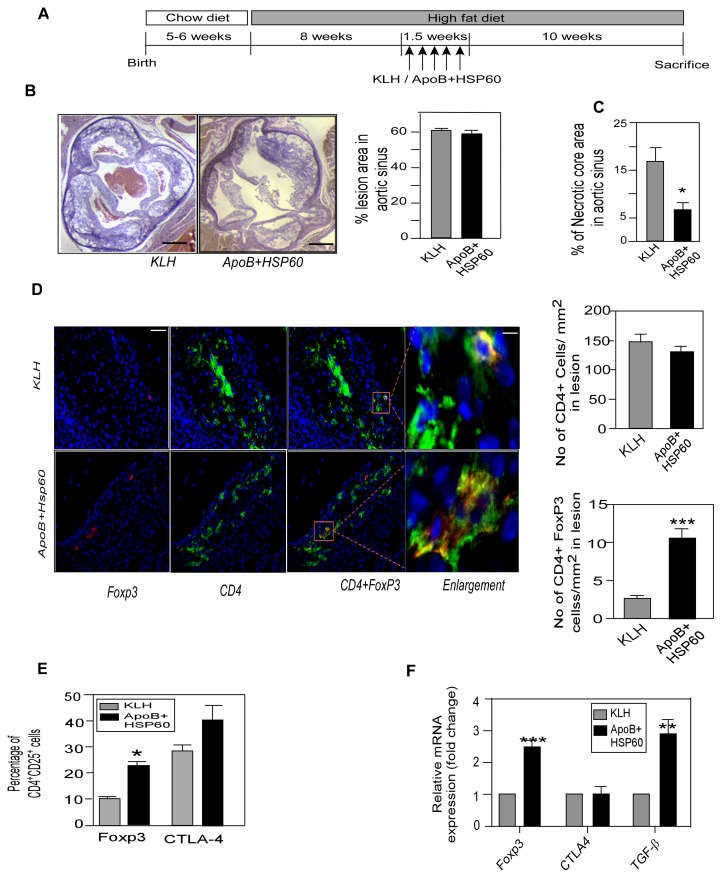
Oral Tolerance with Continued Hypercholesterolemia Stabilizes Vulnerable Plaque. A. Experimental design. B. Representative photomicrographs of plaque area stained with EVG and its quantitative analysis in aortic sinus of 26 week old ApoB^tm25gy^LDLr^tm1Her^ mice (n = 10 per group). Scale bar represents 200 µm C. Percentage of acellular necrotic core area in total plaque area. *P = 0.012 D. Representative photomicrographs showing double immunofluorescence staining of aortic sinus sections with CD4 (green) and Foxp3 (red). Scale bar represents 50 µm. Enlarged region to show double immune staining. Scale bar represents 6.25 µm. Right panel: Number of CD4-positive cells/mm^2^ and CD4+ Foxp3+ cells/mm^2^ (n = 9per group). ***P<0.001. E. Percentage of CD25^+^Foxp3^+^ cells (*P<0.009) and CTLA-4 (NS) within the CD4 population in spleen (n = 6 per group). F. Expression of mRNA of Foxp3 (***P = 0.003), CTLA-4 (P = NS), and TGF-β (**P = 0.005) in the ascending aorta quantified by RT-PCR and normalized to GAPDH. Fold-changes in their expression in ApoB+HSP60-tolerized mice relative to controls (n = 4 per group).

To study the effect of tolerance on advanced lesions, ApoB/LDLr^−/−^ mice were fed a high-fat diet for 10 weeks, to establish lesion, orally dosed with peptides or KLH in the last 1.5 weeks, and continued this diet for the next 10 weeks (Figure 5A). Cholesterol concentrations in the plasma increased to 20.98±1.67 mMol/L for KLH and 21.16±1.59 mMol/L for peptide-tolerized mice (Table S1B). Both groups showed an increase in lesion area in the aortic sinus (61.03±1.61% and 58.69±1.40%, respectively) (Figures 5B and S5). Close observation of the aortic sinus revealed a 60.8% reduction in the percentage of necrotic core areas in peptide-tolerized mice compared with controls (6.5±1.4% vs. 16.6±2.9%, P = 0.012) (Figure 5C).

We then examined the effect of oral dosing on regulatory T cells in advanced atherosclerosis. The number of CD4^+^ cells expressing Foxp3^+^ regulatory T-cell markers were higher (10.5±1.3% vs. 2.6±0.2%, P<0.001) in the aortic sinus of the peptide-tolerized mice, compared with the controls (Figure 5D). Flow cytometry analysis of splenocytes also indicated a significant increased expression of Foxp3 in CD4^+^CD25^+^ cells in spleen (P = 0.009), but the increase in CTLA-4 expression was not significant (Figure 5E, and S6A). Consistent with these results, we observed an increase in Foxp3 (2.5-fold), TGF-β (3 fold) expression in the aorta by RT-PCR analysis, but CTLA-4 expression was comparable between the groups (Figure 5F).Plasma TGF-β concentrations were not found be significantly different (Figure S6B).

### Plaque Vulnerability Markers are Reduced in ApoB+HSP60 Peptide Tolerized Mice

**Figure 6:**
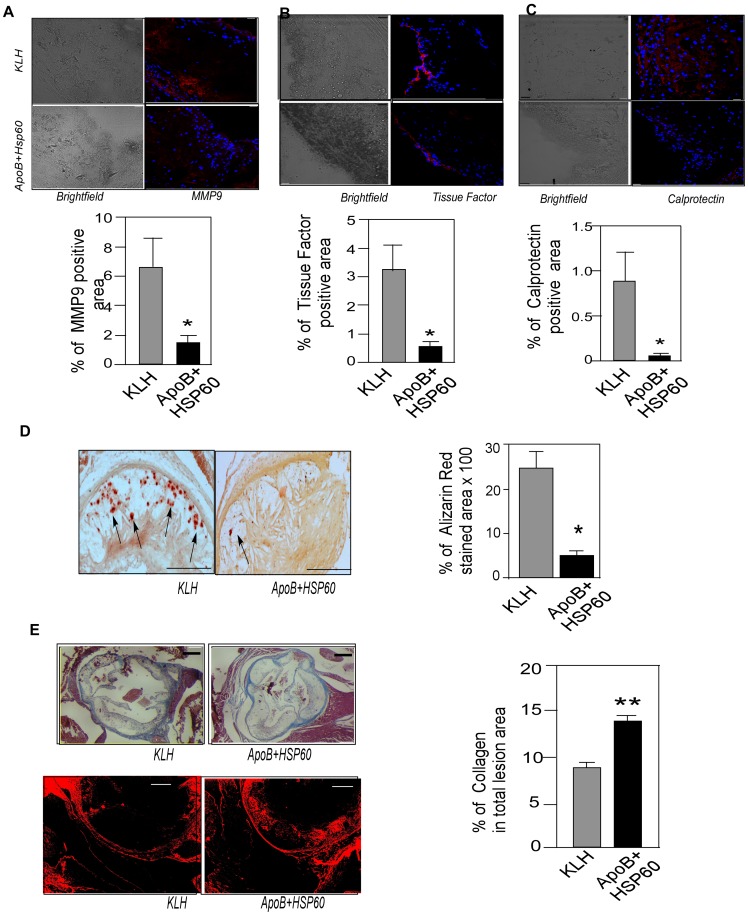
Reduction in Plaque Vulnerability Markers in Tolerized Mice. A. Representative photomicrographs showing immunofluorescence staining of aortic sinus sections with MMP9 (red) and its quantitative analysis (n = 10 per group). *P = 0.035 B. Representative photomicrographs showing immunofluorescence staining of aortic sinus sections with tissue factor (red) and its quantitative analysis (n = 6 per group). *P = 0.028 C. Representative photomicrographs showing immunofluorescence staining of aortic sinus sections with calprotectin (MRP8/14) (red) and its quantitative analysis (n = 6 per group). *P = 0.045 D. Representative photomicrographs of aortic sinus sections stained with alizarin-red S. (n = 6 per group) Arrows indicate calcium depositions and its quantitative analysis. *P = 0.018. Scale bar represents 150 µm. E. Representative photomicrographs of aortic sinus sections stained with Masson’s trichrome and its quantitative analysis (n = 8 per group). **P = 0.002. Lower photomicrograph shows picrosirius staining for collagen content Scale bar represents 200 µm. Scale bar represents 50 µm for the immunofluorescence staining.

Next we examined the markers of plaque vulnerability in aortic sinus from both groups of mice by immunohistochemistry to correlate their expression with plaque stability. Expression of MMP9 (P = 0.035), tissue factor (P = 0.028), and Mrp8/14 (P = 0.045) was significantly lower in peptide-tolerized mice compared with controls (Figure 6A–C). Extensive calcification, as seen by Alizarin red staining was observed in KLH-control mice,while it was 80% lower in peptide tolerized mice (P = 0.018) (Figure 6D). In contrast, collagen staining using Masson’s trichrome as well as picrosirius-red revealed increased plaque collagen content peptide-tolerized mice compared with KLH- control, suggesting features of plaque stabilization in these animals (Figure 6E).

### Plaque Stabilization is Associated with Reduced Apoptosis

**Figure 7:**
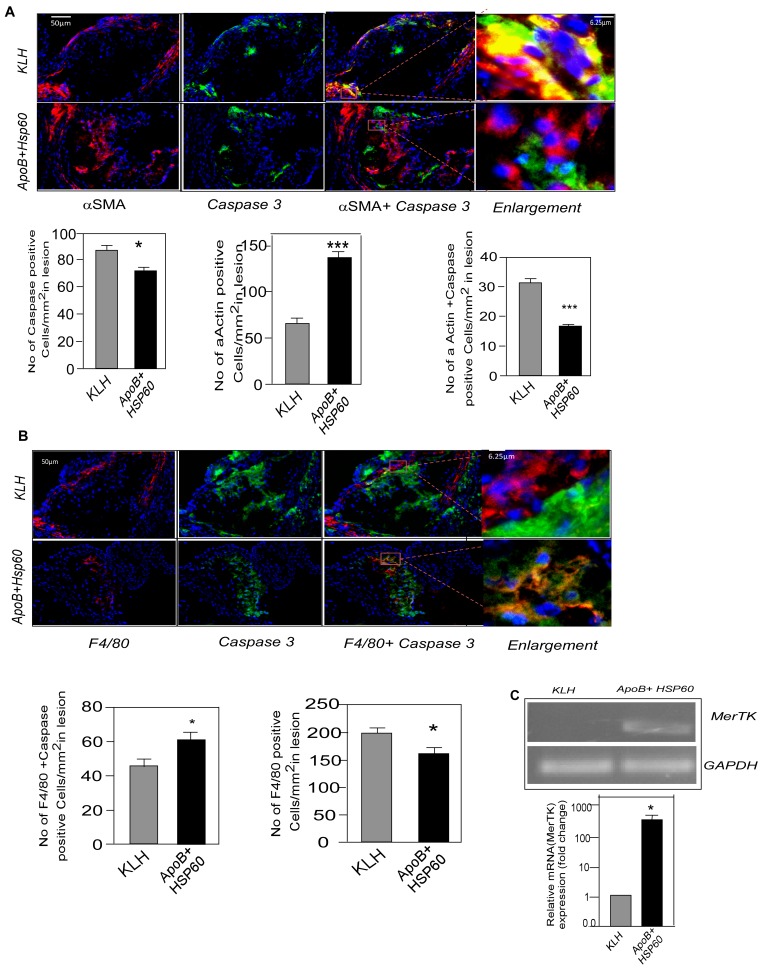
Plaque Stabilization Associated with Reduced Apoptosis. A. Representative photomicrographs showing double immunofluorescence staining of aortic sinus sections with αSMA (red) and Caspase 3 (green). Scale bar represents 50 µm. Enlarged region to show double immune staining. Scale bar represents 6.25 µm. Number of Caspase 3 positive cells/mm^2^ (n = 12). *P = 0.017.Number of αSMA-positive smooth muscle cells/mm^2^ (n = 11) (***P<0.001) and αSMA^+^Caspase^+^ cells/mm^2^ (n = 11 per group). ***P<0.001 for APOB+HSP60 tolerized animals compared with KLH. B. Representative photomicrographs showing double immunofluorescence staining of aortic sinus sections with F4/80 (red) and Caspase 3 (green). Scale bar represents 50 µm. Enlarged region to show double immune staining. Scale bar represents 6.25 µm. Number of F4/80 -positive macrophages/mm^2^ (n = 9 per group) * P = 0.02 for APOB+HSP60 tolerized animals compared to KLH and F4/80^+^ Caspase^+^ cells/mm^2^ (n = 9 per group). *P = 0.03 for KLH compared to APOB+HSP60 tolerized animals. C. Expression of MerTK mRNA in the ascending aorta quantified by RT-PCR analysis and normalized to GAPDH. Fold-changes in the expression in ApoB+HSP60-tolerized mice relative to controls (n = 4 per group). *P = 0.029.

To understand the possible mechanism of plaque stabilization following oral tolerance, we studied extent of apoptosis in the advanced aortic lesions by immunohistochemical analysis. The number of apoptotic cells as seen by Caspase3 staining were significantly lower in peptide tolerized mice compared to control (86.38 4.5 *vs.* 71.55 3.48, p = 0.017) (Figure 7A). We also observed a reduction in TUNEL positivity in the peptide-tolerized mice (Figure S7).We then examined the nature of cells which are undergoing apoptosis in the advanced lesion by double immuno staining for macrophages and smooth muscle cells (SMC) along with anti caspase3 antibodies. Apoptosis of SMCs was significantly increased in KLH-control mice compared with APOB+HSP60-tolerized animals (31.15±2.97 vs. 16.55±0.95, P<0.001). The number of SMCs were also observed to be higher in ApoB+HSP60 tolerized mice (64.7±6.3 vs. 136.3±6.19, P<0.001) (Figure 7A). To our surprise the number of apoptotic macrophages were higher in tolerized mice (61.36±4.5 vs. 44.9±4.4, P = 0.03), although the number of macrophages was significantly lower in the plaques of peptide-tolerized mice compared with controls (159.9±11.77 vs. 197.7±10.56, P = 0.02) (Figure 7B). We then examined the expression of receptor tyrosine kinase Mer (MerTK) in the lesions by RT–PCR analysis. The expression of MerTK was significantly lower in control mice compared with peptide-tolerized animals, suggesting a defective clearance of apoptotic cells (Figure 7C).

## Discussion

This study presents a new concept that oral tolerance to a combination of ApoB and HSP60 peptides can control progression of atherosclerotic lesions and stabilize unstable plaque.

Several studied have highlighted the importance of oral tolerance in preventing the development of atherosclerosis in animal models. Induction of oral tolerance to oxidized low-density lipoprotein (LDL) in LDL^−/−^ mice was associated with an increase in the number of Foxp3^+^ cells in the spleen and lymph nodes, and increased TGF-β production [23]. Oral administration of HSP60 peptide (253–268) resulted in increase in Treg cells and significant reduction in atherosclerosis in carotid arteries as well as aortic root [24].Recently, we have also shown that oral tolerance to ApoB and HSP 60 peptides can prevent early development of atherosclerosis in mice [28]. Regulatory T-cell response to Apolipoprotein B100–derived peptides was shown to reduce the development and progression of atherosclerosis in ApoE−/− Mice [41]. However the role of tolerance on plaque stability is yet to be established. Therefore we focused on the effect of oral tolerance to autoantigens on plaque regression and its stabilization. We induced tolerance to a combination of ApoB and HSP60 peptides in two experimental groups of hypercholesterolemic mice with established atherosclerotic plaque. One group was continued on high fat diet while the other was shifted to normal diet to understand the role of tolerance with diet control on plaque progression.

**Figure 8:**
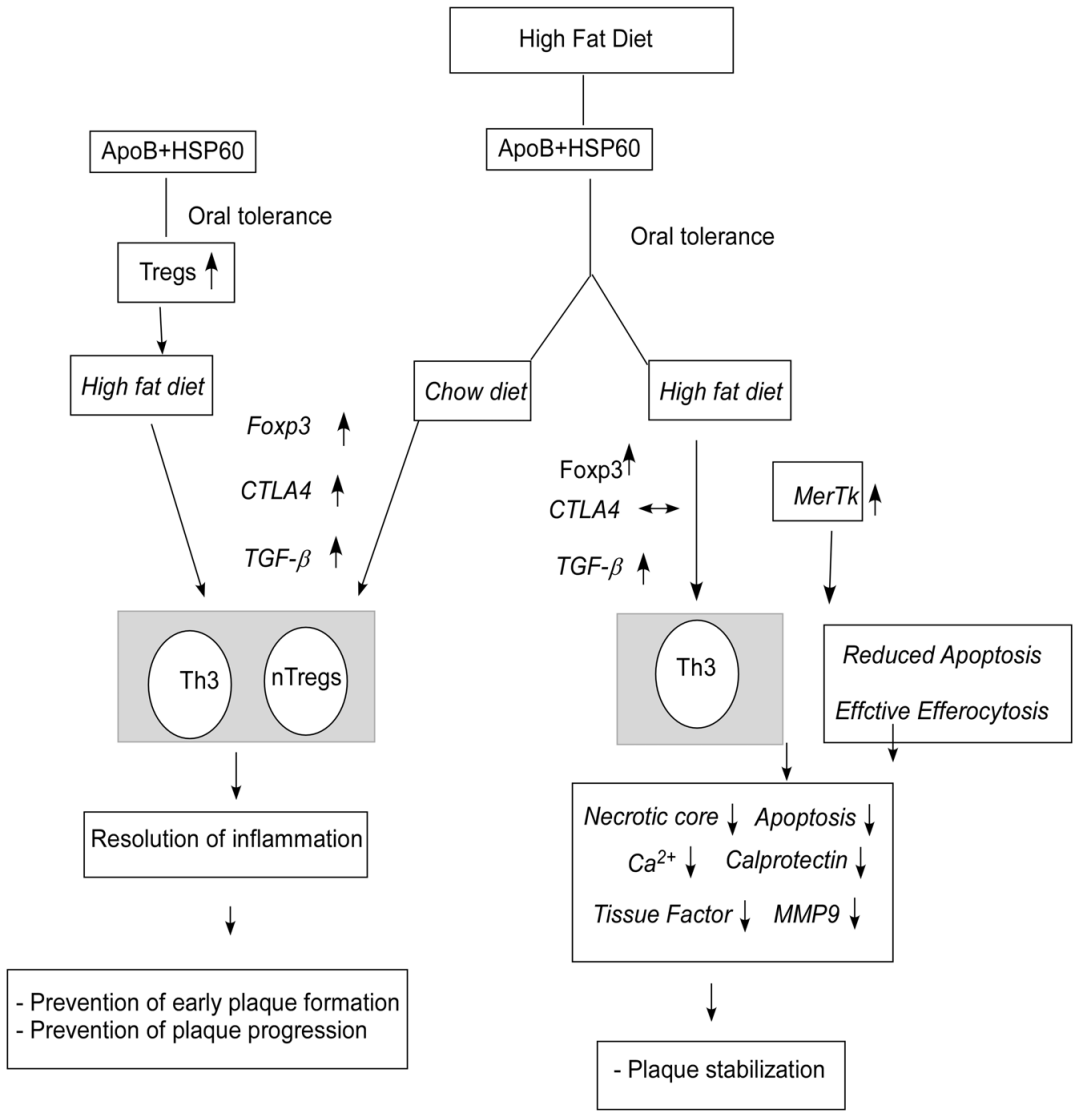
Model Depicting Oral Tolerance-Induced Prevention and Stabilization of Atherosclerotic Lesion Development. Oral administration of peptides (ApoB+HSP60) induces a regulatory T-cell response, which is maintained for 10 weeks and results in prevention of early atherosclerotic lesions. Oral administration of peptides in mice with established lesions in combination with diet modification also induces a Treg-cell response with an increase in CD4+CD25+Foxp3 T cells, CTLA-4 expression, and increased TGF-secretion in the lesion and the peripheral circulation. It is likely that both CTLA-4- and TGF-mediated suppression are required for early lesion reduction and inhibition of plaque progression. With continuous hypercholesterolemia, the CTLA-4 expression remains unaltered, while there is an increase in Foxp3 and TGF expression in tolerized mice. We propose that the TGF-mediated decrease in inflammatory activity results in plaque stabilization.

Oral treatment with peptide epitopes from ApoB and HSP60 induced expansion of Treg cells with antigen-specific suppressor properties for both antigenic peptides. Oral tolerance to the two peptides combined with diet control arrested the progression of lesion development. This was also associated with an increase in CD4^+^CD25^+^Foxp3^+^CTLA-4^+^ Treg cells in the periphery and increased expression of CTLA-4, Foxp3, and TGF-β in the lesion. Recently, Maganto-Garcia et al [42] showed that reversal hypercholesterolemia through a normal diet can prevent the loss of lesional Treg cells, resulting in reduction of lesion progression. Our results support this observation and suggest that diet modification in combination with oral tolerance can prevent the progression of established lesion, most likely mediated by both natural as well as adaptive Th3 Treg cells (Figure 8).

Oral tolerance was unable to control lesion progression with continued hypercholesterolemia, but we observed a considerable reduction in the necrotic core in the aortic sinus. The expression of Foxp3 and TGF-β was higher in the aorta and peripheral lymphocytes of peptide-tolerized mice compared with controls, but no significant up-regulation of CTLA-4 was observed. It is likely that the TGF-β–secreting Tregs, which were induced upon oral immunization, are not sufficient to control lesion progression with continued hypercholesterolemia but play a significant role in stabilizing the plaque.

Recent reports have demonstrated an atheroprotective role of natural Treg (n Treg) cells expressing CD25 and the transcription factor fork head box p3 (Foxp3), which controls the expression of genes associated with regulatory function including cytotoxic T-lymphocyte antigen 4 (CTLA-4) and glucocorticoid-induced tumor necrosis factor receptor [14], [43]. Besides n Treg-adaptive regulatory cells, Tr1 and Th3 cells secreting IL-10 and TGF-β, respectively, have also been implicated in protection against atherogenesis [23], [26], [27]. The mechanisms involved in Treg-mediated suppression of atherosclerosis are still unclear. Does atheroprotection involve contact-dependent suppression, or is it mediated by anti-inflammatory cytokines, or are both required for effective protection? Our results suggest that both types of activity may be required for effective reduction of lesion development. The atheroprotective effect of oral tolerance to the combination of HSP60 and ApoB peptides paralleled the induction of CD4^+^CD25^+^Foxp3^+^ Treg cells with immunosuppressive activity against both ApoB100 and HSP60 effector cells. We observed an increase in TGF-β mRNA concentration and protein expression in the lesion, suggesting the role of Th3 cells; and increased expression of CTLA-4 in the lesion and in peripheral CD4 cells, suggesting the role of contact-dependent suppression in reducing early lesion formation (Figure 8). TGF-β is a potent anti-atherogenic cytokine and a key molecule contributing to peripheral tolerance [44]. Recent reports suggest that TGF-β–producing Th3 cells play a crucial role in inducing and maintaining peripheral tolerance by helping the differentiation of antigen-specific Foxp3+ cells in the periphery [45]. TGF-β can suppress the recruitment of macrophages into the lesion, uptake of oxidized LDL, formation of foam cells, and can activate Treg cells, phagocytosis, and collagen biosynthesis, thus resolving inflammation [46], [47].

Plaque necrosis in advanced atheroma is caused by apoptosis of macrophages, T lymphocytes, and smooth muscle cells (SMCs), coupled with defective phagocytic clearance of dead cells [48], [49]. Macrophages can trigger the apoptosis of SMCs in vulnerable plaques by secreting pro-apoptotic cytokines including TNF-α and the release of reactive oxygen species [50]. Apoptotic cells are normally phagocytosed by dendritic cells and macrophages, which in turn inhibit the production of proinflammatory cytokines involving the secretion of TGF-β [51]. Defective clearance of apoptotic cells (or efferocytosis) results in a persistent inflammatory state, causing the formation of the necrotic core or vulnerable plaque [52]. Prolonged failure of efferocytosis leads to post-apoptotic secondary necrosis and the release of matrix proteases and tissue factor, which can amplify the inflammation. MerTK is a tyrosine kinase receptor for the phosphatidylserine-binding protein Gas6, which bridges apoptotic cells to phagocytes [53]. Absence of the MerTK receptor reduces the efficiency of efferocytosis and promotes apoptotic cell accumulation and plaque necrosis in atherosclerotic lesions of ApoE^−/−^ mice [54]. In the present study we observed a higher number of SMCs in the plaque, a decrease in their apoptosis in association with plaque stabilization, and a 400-fold higher expression of MerTK in the aorta of tolerized mice, suggesting that efficient efferocytosis prevents accumulation of apoptotic cells in the lesion, which in turn resolves plaque inflammation. Despite higher expression of MerTK, we observed a higher percentage of apoptotic macrophages in the lesions of peptide-tolerized mice, but a significant reduction in apoptotic SMCs. A recent study by Stoneman *et al* [55] suggests that suggests that macrophages can be cleared from atherosclerotic plaques in a clean and safe way via selective induction of macrophage apoptosis. It is likely that the macrophages are effectively cleared in the peptide-tolerized mice and thus prevent secondary necrosis by inducing apoptosis in SMCs.

One possible mechanism by which oral immunization could have stabilized the lesion may be mediated by TGF-β. Indeed, we found a higher level of TGF-β mRNA in the aorta of tolerized mice. The expression of CTLA4 was similar to that in controls, suggesting a defect in contact-mediated pathogenic effector-cell suppression. TGF-β-mediated efferocytosis and collagen synthesis by fibroblasts could possibly have prevented the uncontrolled inflammation and stabilization of plaque, though this was not sufficient to reduce the progression. Thus oral immunization with a combination of ApoB and HSP60 peptides attenuates atherosclerotic lesion formation and its progression, which is associated with an increase in numbers of nTreg and Th3 cells. These cells are probably generated in gut-associated lymphoid organs and migrate to other peripheral lymphoid tissues and then to the atherosclerotic plaque, leading to suppression of the pathogenic T-cell response and promoting an anti-inflammatory milieu with an atheroprotective effect.

Atherosclerosis starts at an early age in humans and is a slow progressing disease.

Vaccination would be a successful approach to treat the disease provided that it is effective at various stages of the disease process. Our data suggest that oral tolerance to multiple self-epitopes can stabilize the growing plaque and control plaque progression in conjunction with diet control. No detectable difference was observed in antibody levels to the peptides, as reported in earlier studies with oral tolerance to oxidized LDL and HSP60 [23], [24]. Further studies directed at refining the protocol for tolerance induction, developing appropriate formulations and evaluating efficacy in different animal models and understanding the mechanism of protection are required to establish oral tolerance as a successful immune therapy for atherosclerosis.

## Supporting Information

Figure S1 **Quantification of atherosclerosis in descending aorta in ApoB+ HSP60 peptides tolerized mice compared to control.** A. Enface staining. Left panels: En-face staining: whole aortas were collected in NBF and used for en-face analysis using Oil red-O staining. Right panel: Lesion area of the aorta was quantified relative to its surface area using Image-Pro Plus software (n = 4 per group). B. Comparison of lesion area in PBS and KLH-treated animals. Left panels: Representative photomicrographs of aortic sinus plaque area stained with EVG (hearts were sectioned at the end of the study). Scale bar represents 200 µm. Right panel: Percentage of plaque area in total aortic sinus. Right panel: Percentage of plaque area in total aortic sinus (n = 6 per group).
(TIF)

Figure S2 **Flow cytometry analysis of splenocytes and plasma cytokine concentrations following oral tolerance to peptides.** Splenocytes was prepared at week 20, after 10 weeks of a high-fat diet following oral tolerance induction. A. Representative FACS dot plots showing CD4, CD4+ Foxp3+ and CD4+ CTLA4+from spleen are presented (n = 6 per group). B. Percentage of CD4+ CD25+Foxp3+ and CD4+ CTLA4+ cells in lymph nodes. C. Percentage of CD4+ CD25+Foxp3+ and CD4+ CTLA4+ cells in peripheral blood. D. Splenocytes were stimulated with concavalin A (10 ug/mL) in vitro for 72 h. Cytokine concentrations in the supernatant were estimated by ELISA (n = 6 per group). *P = 0.017 for TGF-β concentration. E. Plasma cytokine concentrations: IFN-γ levels in the plasma of mice tolerized to peptides and control (n = 6) for each group, measured by ELISA. *P = 0.02. F. TGF-β concentration in the plasma (n = 6) for each group measured by ELISA. *P = 0.01.
(TIF)

Figure S3 **Quantification of atherosclerosis in descending aorta in mice tolerized to ApoB+HSP60 peptides in Combination with Diet Modification.** Mice were fed with high fat diet to establish lesion, orally dosed with KLH as control or peptides (ApoB+HSP60) and shifted to chow diet at the end of 10 weeks. A. Comparison of KLH and PBS control for lesion development: Representative photomicrographs of EVG stained plaque area and its quantitative analysis in aortic sinus of 26 week old ApoB^tm25gy^LDLr^tm1Her^ mice. Scale bar represents 200 µm. Right panel: Percentage of plaque area in total aortic sinus. Right panel: Percentage of plaque area in total aortic sinus (n = 6 per group). B. En-face analysis of the aorta from mice immunized orally with peptides (ApoB+HSP60) or KLH, after establishment of lesion, Left panel: Whole aortas were collected in NBF and used for en-face analysis using Oil red-O staining. Right panel: Lesion area of the aorta was quantified relative to its surface area using ImagePro Plus software (n = 3 per group).
(TIF)

Figure S4 **Flow cytometry analysis of splenocytes and plasma cytokine concentrations following oral tolerance to peptides in combination with diet modification.** A. Flow cytometry analysis of lymphocytes from splenocytes. Spleen cells were prepared from mice immunized orally with peptides (ApoB+HSP60) or KLH, after establishment of lesion, and fed a normal chow diet following tolerance induction. Representative FACS dot plots showing CD4^+^, CD4^+^ Foxp3^+^ and CD4^+^ CTLA4^+^ from spleen are presented (n = 6 per group). B. Plasma concentrations of TGF-β (n = 6). *P = 0.02.
(TIF)

Figure S5 **Quantification of atherosclerosis in descending aorta in mice tolerized to ApoB+HSP60 peptides with continued hyperlipidaemia.** En-face analysis of the aorta from mice immunized orally with peptides (ApoB+HSP60) or KLH, after establishment of lesion, and continued on a high-fat diet following tolerance induction. Left panel. Whole aortas were collected in NBF and used for en-face analysis using Oil red-O staining. Right panel. Lesion area of the aorta was quantified relative to its surface area using Image Pro Plus software (n = 4 per group). Left panels: Representative photomicrographs of brachiocephalic plaque area stained with EVG (hearts were sectioned at the end of the study). Scale bar represents 200 µm. Right panel: Percentage of plaque area in total brachiocephalic artery, *P = 0.001 for A+H tolerized.
(TIF)

Figure S6 **Flow cytometry analysis of splenocytes and plasma cytokine concentrations following oral tolerance to peptides with continued hyperlipidaemia.** A. Flow cytometry analysis of lymphocytes from splenocytes was prepared from mice immunized orally with peptides (ApoB+HSP60) or KLH, after establishment of lesion, and continued on a high-fat diet following tolerance induction. Representative FACS dot plots showing CD4^+^, CD4^+^ Foxp3^+^ and CD4^+^ CTLA4^+^ from spleen cells are presented (n = 6 per group). B. Plasma concentrations of TGF-β (n = 6). P = NS.
(TIF)

Figure S7 **TUNEL Assay.** Apoptosis as studied by TUNEL assay in aorta from mice immunized orally with peptides (ApoB+HSP60) or KLH, after establishment of lesion, and continued on a high-fat diet following tolerance induction. Aortic sections stained with TMR red (Roche Applied Science) as described by the manufacturers instructions and imaged using confocal microscope. Scale bars represent 50 µm.
(TIF)

Table S1 **Plasma lipid levels.**
(DOCX)

Table S2 **Antibody response to peptides.**
(DOCX)

Method S1 **Terminal deoxynucleotidyl transferase dUTP nick end labeling (TUNEL).**
(DOC)

Method S2 **Antibody response measurement.**
(DOC)

Method S3 **Real-time reverse transcription polymerase chain reaction (RT-PCR) analysis.**
(DOC)

## References

[1] Hansson GK (2005) Inflammation, atherosclerosis, and coronary artery disease. N Engl J Med 352: 1685–1695. 15843671 10.1056/NEJMra043430

[2] Hansson GK (2009) Atherosclerosis–an immune disease: The Anitschkov Lecture 2007. Atherosclerosis 202: 2–10. 18951547 10.1016/j.atherosclerosis.2008.08.039

[3] Binder CJ, Chang MK, Shaw PX, Miller YI, Hartvigsen K, et al (2002) Innate and acquired immunity in atherogenesis. Nat Med 8: 1218–1226. 12411948 10.1038/nm1102-1218

[4] Hansson GK (2001) Immune mechanisms in atherosclerosis. Arterioscler Thromb Vasc Biol 21: 1876–1890. 11742859 10.1161/hq1201.100220

[5] Grundtman C, Kreutmayer SB, Almanzar G, Wick MC, Wick G (2011) Heat shock protein 60 and immune inflammatory responses in atherosclerosis. Arterioscler Thromb Vasc Biol 31: 960–968. 21508342 10.1161/ATVBAHA.110.217877 PMC3212728

[6] Wick G, Knoflach M, Xu Q (2004) Autoimmune and inflammatory mechanisms in atherosclerosis. Annu Rev Immunol 22: 361–403. 15032582 10.1146/annurev.immunol.22.012703.104644

[7] Nilsson J, Hansson GK (2008) Autoimmunity in atherosclerosis: a protective response losing control? J Intern Med 263: 464–478. 18410590 10.1111/j.1365-2796.2008.01945.x

[8] Grundtman C, Wick G (2011) The autoimmune concept of atherosclerosis. Curr Opin Lipidol 22: 327–334. 21881502 10.1097/MOL.0b013e32834aa0c2 PMC3216126

[9] Almanzar G, Ollinger R, Leuenberger J, Onestingel E, Rantner B, et al.. (2012) Autoreactive HSP60 epitope-specific T-cells in early human atherosclerotic lesions. J Autoimmun. 10.1016/j.jaut.2012.07.006 PMC3516706 22901435

[10] Tabas I, Williams KJ, Boren J (2007) Subendothelial lipoprotein retention as the initiating process in atherosclerosis: update and therapeutic implications. Circulation 116: 1832–1844. 17938300 10.1161/CIRCULATIONAHA.106.676890

[11] Sakaguchi S (2005) Naturally arising Foxp3-expressing CD25+CD4+ regulatory T cells in immunological tolerance to self and non-self. Nat Immunol 6: 345–352. 15785760 10.1038/ni1178

[12] von Boehmer H (2005) Mechanisms of suppression by suppressor T cells. Nat Immunol 6: 338–344. 15785759 10.1038/ni1180

[13] Mallat Z, Tedgui A (2004) Immunomodulation to combat atherosclerosis: the potential role of immune regulatory cells. Expert Opin Biol Ther 4: 1387–1393. 15335306 10.1517/14712598.4.9.1387

[14] Mallat Z, Ait-Oufella H, Tedgui A (2007) Regulatory T-cell immunity in atherosclerosis. Trends Cardiovasc Med 17: 113–118. 17482092 10.1016/j.tcm.2007.03.001

[15] Nilsson J, Bjorkbacka H, Fredrikson GN (2012) Apolipoprotein B100 autoimmunity and atherosclerosis - disease mechanisms and therapeutic potential. Curr Opin Lipidol 23: 422–428. 22814703 10.1097/MOL.0b013e328356ec7c

[16] Nilsson J, Fredrikson GN, Bjorkbacka H, Chyu KY, Shah PK (2009) Vaccines modulating lipoprotein autoimmunity as a possible future therapy for cardiovascular disease. J Intern Med 266: 221–231. 19702790 10.1111/j.1365-2796.2009.02150.x

[17] Binder CJ, Hartvigsen K, Witztum JL (2007) Promise of immune modulation to inhibit atherogenesis. J Am Coll Cardiol 50: 547–550. 17678739 10.1016/j.jacc.2007.04.054

[18] Binder CJ, Horkko S, Dewan A, Chang MK, Kieu EP, et al (2003) Pneumococcal vaccination decreases atherosclerotic lesion formation: molecular mimicry between Streptococcus pneumoniae and oxidized LDL. Nat Med 9: 736–743. 12740573 10.1038/nm876

[19] Gaofu Q, Jun L, Xiuyun Z, Wentao L, Jie W, et al (2005) Antibody against cholesteryl ester transfer protein (CETP) elicited by a recombinant chimeric enzyme vaccine attenuated atherosclerosis in a rabbit model. Life Sci 77: 2690–2702. 15963532 10.1016/j.lfs.2005.05.037

[20] Holmgren J, Czerkinsky C (2005) Mucosal immunity and vaccines. Nat Med 11: S45–53. 15812489 10.1038/nm1213

[21] Weiner HL, da Cunha AP, Quintana F, Wu H (2011) Oral tolerance. Immunol Rev 241: 241–259. 21488901 10.1111/j.1600-065X.2011.01017.x PMC3296283

[22] Maron R, Sukhova G, Faria AM, Hoffmann E, Mach F, et al (2002) Mucosal administration of heat shock protein-65 decreases atherosclerosis and inflammation in aortic arch of low-density lipoprotein receptor-deficient mice. Circulation 106: 1708–1715. 12270867 10.1161/01.cir.0000029750.99462.30

[23] van Puijvelde GH, Hauer AD, de Vos P, van den Heuvel R, van Herwijnen MJ, et al (2006) Induction of oral tolerance to oxidized low-density lipoprotein ameliorates atherosclerosis. Circulation 114: 1968–1976. 17060383 10.1161/CIRCULATIONAHA.106.615609

[24] van Puijvelde GH, van Es T, van Wanrooij EJ, Habets KL, de Vos P, et al (2007) Induction of oral tolerance to HSP60 or an HSP60-peptide activates T cell regulation and reduces atherosclerosis. Arterioscler Thromb Vasc Biol 27: 2677–2683. 17901374 10.1161/ATVBAHA.107.151274

[25] George J, Yacov N, Breitbart E, Bangio L, Shaish A, et al (2004) Suppression of early atherosclerosis in LDL-receptor deficient mice by oral tolerance with beta 2-glycoprotein I. Cardiovasc Res. 62: 603–609. 10.1016/j.cardiores.2004.01.028 15158153

[26] Sasaki N, Yamashita T, Takeda M, Shinohara M, Nakajima K, et al (2009) Oral anti-CD3 antibody treatment induces regulatory T cells and inhibits the development of atherosclerosis in mice. Circulation 120: 1996–2005. 19884470 10.1161/CIRCULATIONAHA.109.863431

[27] Klingenberg R, Lebens M, Hermansson A, Fredrikson GN, Strodthoff D, et al (2010) Intranasal immunization with an apolipoprotein B-100 fusion protein induces antigen-specific regulatory T cells and reduces atherosclerosis. Arterioscler Thromb Vasc Biol 30: 946–952. 20167655 10.1161/ATVBAHA.109.202671

[28] Mundkur L, Mukhopadhyay R, Deshpande V, Samson S, Tarate S, et al (2013) Comparison of Oral Tolerance to ApoB and HSP60 Peptides in Preventing Atherosclerosis Lesion Formation in Apob48−/Ldlr- Mice. Journal of Vaccines 2013: 13.

[29] Altwegg LA, Neidhart M, Hersberger M, Muller S, Eberli FR, et al (2007) Myeloid-related protein 8/14 complex is released by monocytes and granulocytes at the site of coronary occlusion: a novel, early, and sensitive marker of acute coronary syndromes. Eur Heart J 28: 941–948. 17387139 10.1093/eurheartj/ehm078

[30] Farb A, Burke AP, Tang AL, Liang TY, Mannan P, et al (1996) Coronary plaque erosion without rupture into a lipid core. A frequent cause of coronary thrombosis in sudden coronary death. Circulation 93: 1354–1363. 8641024 10.1161/01.cir.93.7.1354

[31] Virmani R, Burke AP, Kolodgie FD, Farb A (2002) Vulnerable plaque: the pathology of unstable coronary lesions. J Interv Cardiol 15: 439–446. 12476646 10.1111/j.1540-8183.2002.tb01087.x

[32] Thorp E, Li G, Seimon TA, Kuriakose G, Ron D, et al (2009) Reduced apoptosis and plaque necrosis in advanced atherosclerotic lesions of Apoe−/− and Ldlr−/− mice lacking CHOP. Cell Metab 9: 474–481. 19416717 10.1016/j.cmet.2009.03.003 PMC2695925

[33] Dickhout JG, Lhotak S, Hilditch BA, Basseri S, Colgan SM, et al (2011) Induction of the unfolded protein response after monocyte to macrophage differentiation augments cell survival in early atherosclerotic lesions. FASEB J 25: 576–589. 20966213 10.1096/fj.10-159319

[34] Lu X, Chen D, Endresz V, Xia M, Faludi I, et al (2010) Immunization with a combination of ApoB and HSP60 epitopes significantly reduces early atherosclerotic lesion in Apobtm2SgyLdlrtm1Her/J mice. Atherosclerosis 212: 472–480. 20609438 10.1016/j.atherosclerosis.2010.06.007

[35] Fredrikson GN, Schiopu A, Berglund G, Alm R, Shah PK, et al (2007) Autoantibody against the amino acid sequence 661–680 in apo B-100 is associated with decreased carotid stenosis and cardiovascular events. Atherosclerosis 194: e188–192. 17214995 10.1016/j.atherosclerosis.2006.12.014

[36] Farese RV Jr, Veniant MM, Cham CM, Flynn LM, Pierotti V, et al (1996) Phenotypic analysis of mice expressing exclusively apolipoprotein B48 or apolipoprotein B100. Proc Natl Acad Sci U S A 93: 6393–6398. 8692825 10.1073/pnas.93.13.6393 PMC39033

[37] Feng B, Zhang D, Kuriakose G, Devlin CM, Kockx M, et al (2003) Niemann-Pick C heterozygosity confers resistance to lesional necrosis and macrophage apoptosis in murine atherosclerosis. Proceedings of the National Academy of Sciences 100: 10423–10428. 10.1073/pnas.1732494100 PMC193577 12923293

[38] Vikramadithyan RK, Hu Y, Noh HL, Liang CP, Hallam K, et al (2005) Human aldose reductase expression accelerates diabetic atherosclerosis in transgenic mice. J Clin Invest 115: 2434–2443. 16127462 10.1172/JCI24819 PMC1190371

[39] Boks MA, Zwaginga JJ, van Ham SM, ten Brinke A (2010) An optimized CFSE-based T-cell suppression assay to evaluate the suppressive capacity of regulatory T-cells induced by human tolerogenic dendritic cells. Scand J Immunol 72: 158–168. 20618775 10.1111/j.1365-3083.2010.02414.x

[40] Milovanova TN (2007) Comparative analysis between CFSE flow cytometric and tritiated thymidine incorporation tests for beryllium sensitivity. Cytometry B Clin Cytom 72: 265–275. 17328032 10.1002/cyto.b.20166

[41] Herbin O, Ait-Oufella H, Yu W, Fredrikson GN, Aubier B, et al (2012) Regulatory T-cell response to apolipoprotein B100-derived peptides reduces the development and progression of atherosclerosis in mice. Arterioscler Thromb Vasc Biol 32: 605–612. 22223728 10.1161/ATVBAHA.111.242800

[42] Maganto-Garcia E, Tarrio ML, Grabie N, Bu DX, Lichtman AH (2011) Dynamic changes in regulatory T cells are linked to levels of diet-induced hypercholesterolemia. Circulation 124: 185–195. 21690490 10.1161/CIRCULATIONAHA.110.006411 PMC3145407

[43] Ait-Oufella H, Salomon BL, Potteaux S, Robertson AK, Gourdy P, et al (2006) Natural regulatory T cells control the development of atherosclerosis in mice. Nat Med 12: 178–180. 16462800 10.1038/nm1343

[44] Grainger DJ (2004) Transforming growth factor beta and atherosclerosis: so far, so good for the protective cytokine hypothesis. Arterioscler Thromb Vasc Biol 24: 399–404. 14699019 10.1161/01.ATV.0000114567.76772.33

[45] Carrier Y, Yuan J, Kuchroo VK, Weiner HL (2007) Th3 Cells in Peripheral Tolerance. I. Induction of Foxp3-Positive Regulatory T Cells by Th3 Cells Derived from TGF-Î^2^ T Cell-Transgenic Mice. The Journal of Immunology 178: 179–185. 17182553 10.4049/jimmunol.178.1.179

[46] Lutgens E, Gijbels M, Smook M, Heeringa P, Gotwals P, et al (2002) Transforming growth factor-beta mediates balance between inflammation and fibrosis during plaque progression. Arterioscler Thromb Vasc Biol 22: 975–982. 12067907 10.1161/01.atv.0000019729.39500.2f

[47] Mallat Z, Gojova A, Marchiol-Fournigault C, Esposito B, Kamate C, et al (2001) Inhibition of transforming growth factor-beta signaling accelerates atherosclerosis and induces an unstable plaque phenotype in mice. Circ Res 89: 930–934. 11701621 10.1161/hh2201.099415

[48] Bjorkerud S, Bjorkerud B (1996) Apoptosis is abundant in human atherosclerotic lesions, especially in inflammatory cells (macrophages and T cells), and may contribute to the accumulation of gruel and plaque instability. Am J Pathol 149: 367–380. 8701977 PMC1865303

[49] Tabas I, Seimon T, Timmins J, Li G, Lim W (2009) Macrophage apoptosis in advanced atherosclerosis. Ann N Y Acad Sci 1173 Suppl 1E40–45. 19751413 10.1111/j.1749-6632.2009.04957.x PMC2762639

[50] Moore KJ, Tabas I Macrophages in the pathogenesis of atherosclerosis. Cell 145: 341–355. 21529710 10.1016/j.cell.2011.04.005 PMC3111065

[51] Schrijvers DM, De Meyer GR, Herman AG, Martinet W (2007) Phagocytosis in atherosclerosis: Molecular mechanisms and implications for plaque progression and stability. Cardiovasc Res 73: 470–480. 17084825 10.1016/j.cardiores.2006.09.005

[52] Tabas I (2010) Macrophage death and defective inflammation resolution in atherosclerosis. Nat Rev Immunol 10: 36–46. 19960040 10.1038/nri2675 PMC2854623

[53] Scott RS, McMahon EJ, Pop SM, Reap EA, Caricchio R, et al (2001) Phagocytosis and clearance of apoptotic cells is mediated by MER. Nature 411: 207–211. 11346799 10.1038/35075603

[54] Thorp E, Cui D, Schrijvers DM, Kuriakose G, Tabas I (2008) Mertk receptor mutation reduces efferocytosis efficiency and promotes apoptotic cell accumulation and plaque necrosis in atherosclerotic lesions of apoe−/− mice. Arterioscler Thromb Vasc Biol 28: 1421–1428. 18451332 10.1161/ATVBAHA.108.167197 PMC2575060

[55] Stoneman V, Braganza D, Figg N, Mercer J, Lang R, et al (2007) Monocyte/macrophage suppression in CD11b diphtheria toxin receptor transgenic mice differentially affects atherogenesis and established plaques. Circ Res 100: 884–893. 17322176 10.1161/01.RES.0000260802.75766.00 PMC2040259

