# Supplementary figures and images for: Mucosal Tolerance to a Combination of ApoB and HSP60 Peptides Controls Plaque Progression and Stabilizes Vulnerable Plaque in Apobtm2SgyLdlrtm1Her/J Mice
Source: PLoS One. 2013 Mar 11;8(3):e58364. doi: 10.1371/journal.pone.0058364 (PMC3594317; doi:10.1371/journal.pone.0058364)

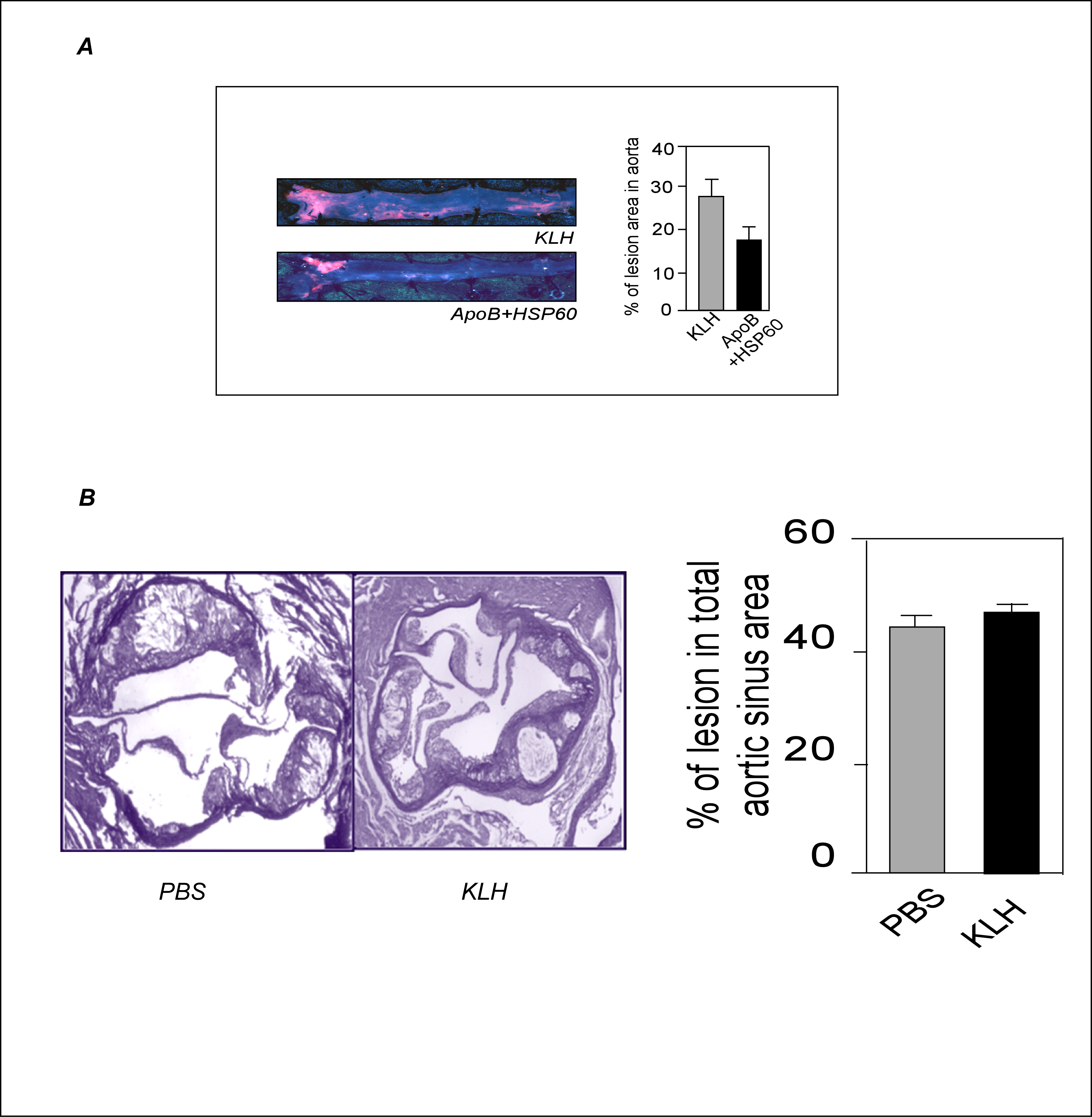

Supplement: Figure S1 — Quantification of atherosclerosis in descending aorta in ApoB+ HSP60 peptides tolerized mice compared to control. A. Enface staining. Left panels: En-face staining: whole aortas were collected in NBF and used for en-face analysis using Oil red-O staining. Right panel: Lesion area of the aorta was quantified relative to its surface area using Image-Pro Plus software (n = 4 per group). B. Comparison of lesion area in PBS and KLH-treated animals. Left panels: Representative photomicrographs of aortic sinus plaque area stained with EVG (hearts were sectioned at the end of the study). Scale bar represents 200 µm. Right panel: Percentage of plaque area in total aortic sinus. Right panel: Percentage of plaque area in total aortic sinus (n = 6 per group). (TIF) [file pone.0058364.s001.tif]

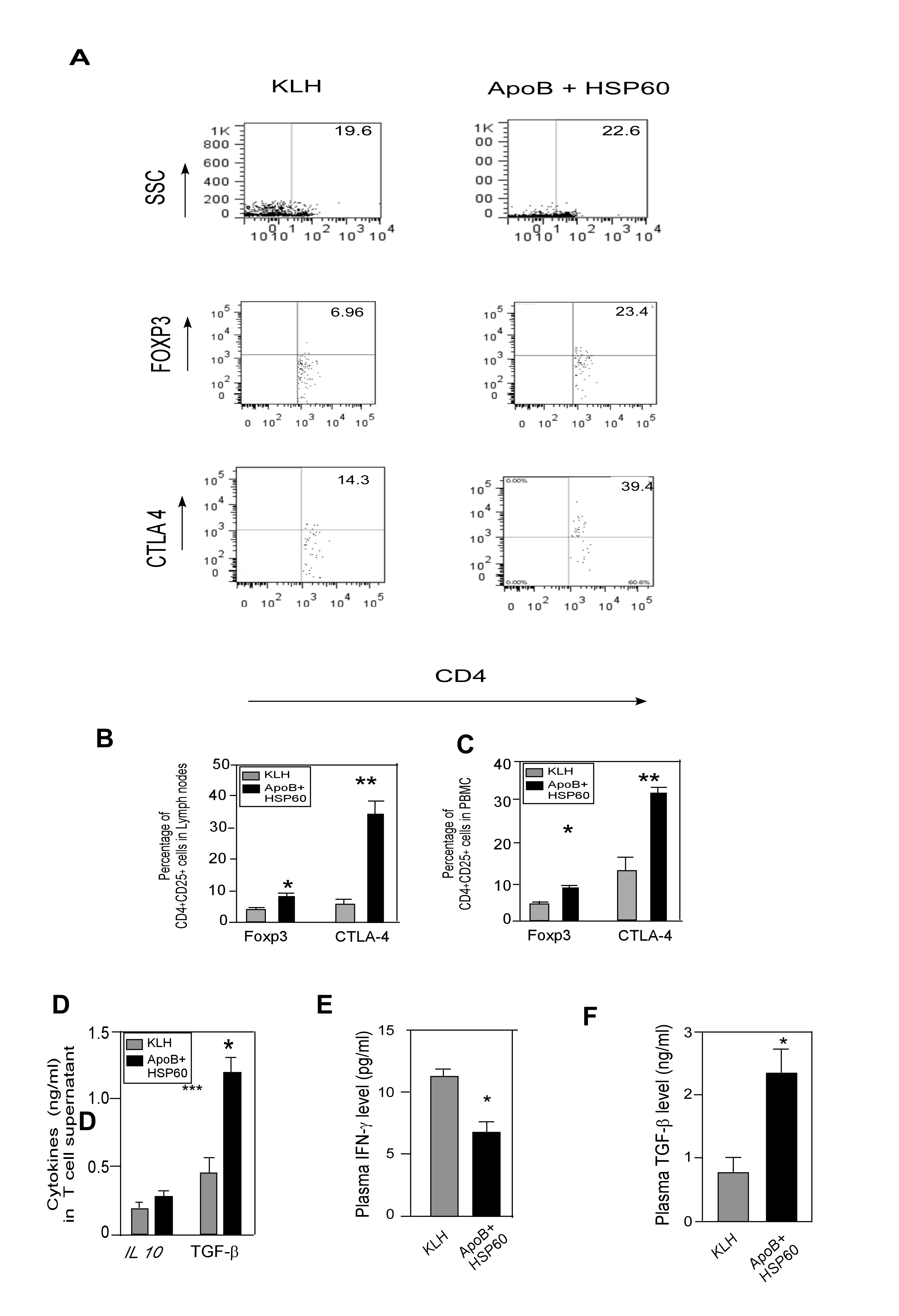

Supplement: Figure S2 — Flow cytometry analysis of splenocytes and plasma cytokine concentrations following oral tolerance to peptides. Splenocytes was prepared at week 20, after 10 weeks of a high-fat diet following oral tolerance induction. A. Representative FACS dot plots showing CD4, CD4+ Foxp3+ and CD4+ CTLA4+from spleen are presented (n = 6 per group). B. Percentage of CD4+ CD25+Foxp3+ and CD4+ CTLA4+ cells in lymph nodes. C. Percentage of CD4+ CD25+Foxp3+ and CD4+ CTLA4+ cells in peripheral blood. D. Splenocytes were stimulated with concavalin A (10 ug/mL) in vitro for 72 h. Cytokine concentrations in the supernatant were estimated by ELISA (n = 6 per group). *P = 0.017 for TGF-β concentration. E. Plasma cytokine concentrations: IFN-γ levels in the plasma of mice tolerized to peptides and control (n = 6) for each group, measured by ELISA. *P = 0.02. F. TGF-β concentration in the plasma (n = 6) for each group measured by ELISA. *P = 0.01. (TIF) [file pone.0058364.s002.tif]

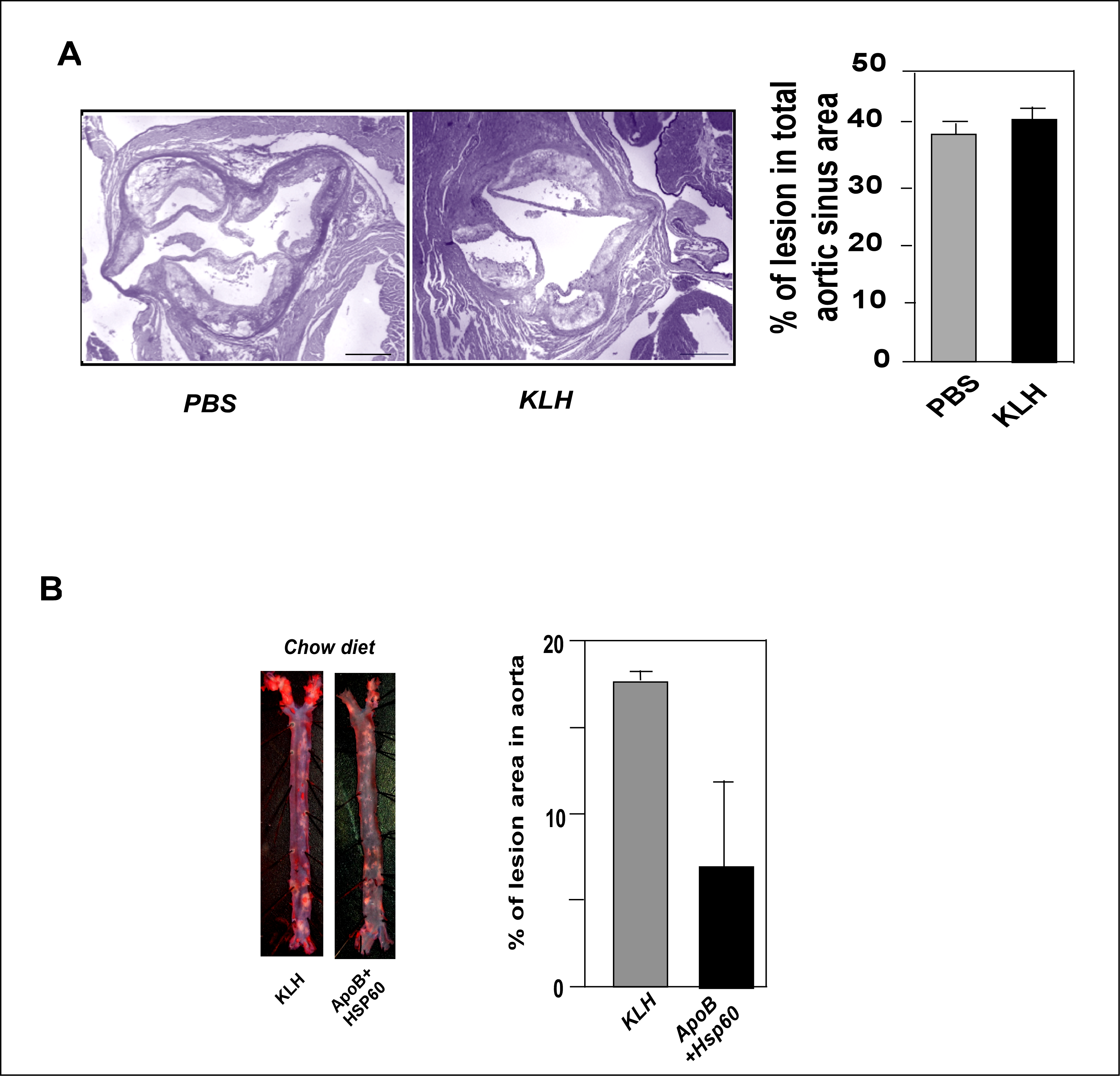

Supplement: Figure S3 — Quantification of atherosclerosis in descending aorta in mice tolerized to ApoB+HSP60 peptides in Combination with Diet Modification. Mice were fed with high fat diet to establish lesion, orally dosed with KLH as control or peptides (ApoB+HSP60) and shifted to chow diet at the end of 10 weeks. A. Comparison of KLH and PBS control for lesion development: Representative photomicrographs of EVG stained plaque area and its quantitative analysis in aortic sinus of 26 week old ApoBtm25gyLDLrtm1Her mice. Scale bar represents 200 µm. Right panel: Percentage of plaque area in total aortic sinus. Right panel: Percentage of plaque area in total aortic sinus (n = 6 per group). B. En-face analysis of the aorta from mice immunized orally with peptides (ApoB+HSP60) or KLH, after establishment of lesion, Left panel: Whole aortas were collected in NBF and used for en-face analysis using Oil red-O staining. Right panel: Lesion area of the aorta was quantified relative to its surface area using ImagePro Plus software (n = 3 per group). (TIF) [file pone.0058364.s003.tif]

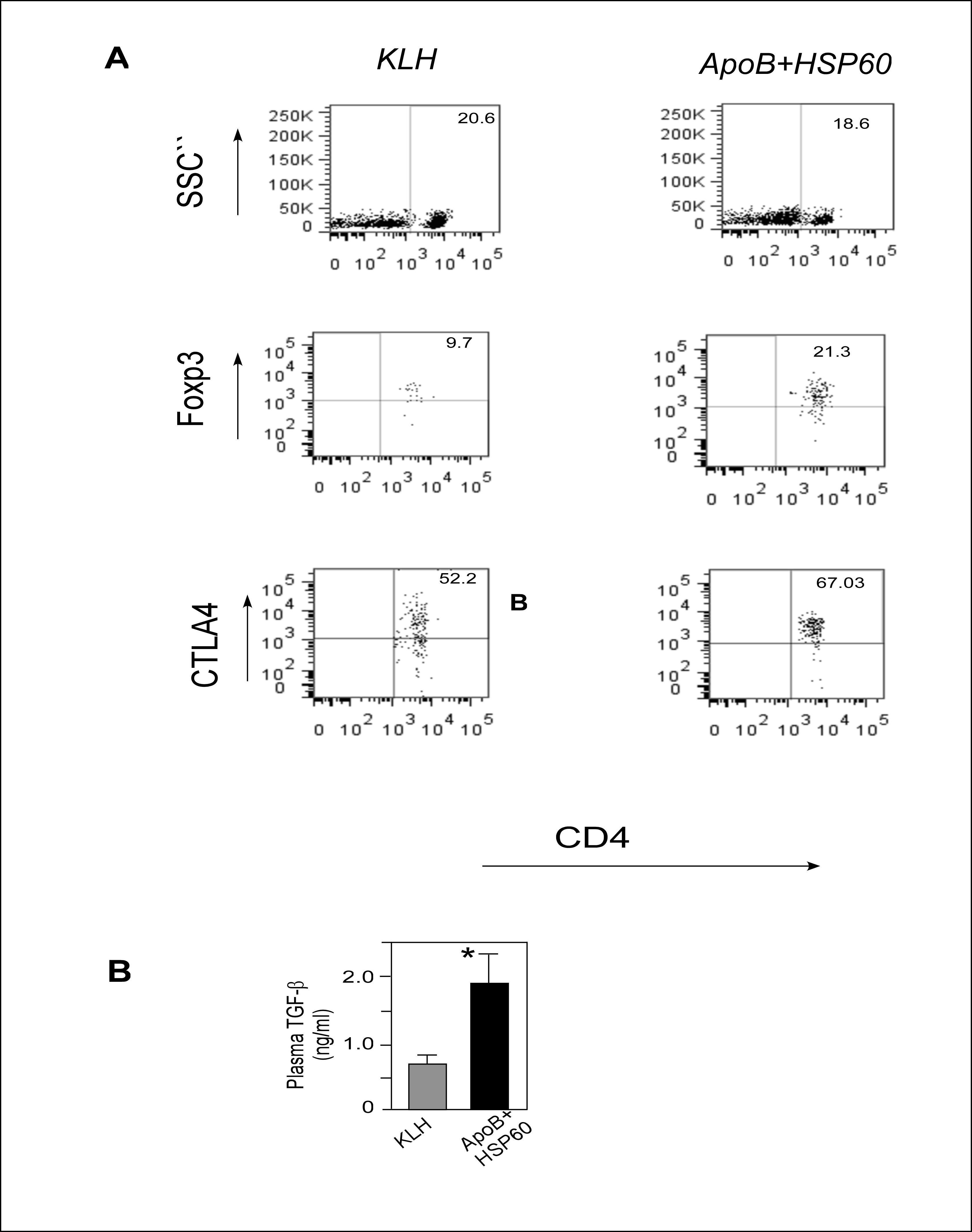

Supplement: Figure S4 — Flow cytometry analysis of splenocytes and plasma cytokine concentrations following oral tolerance to peptides in combination with diet modification. A. Flow cytometry analysis of lymphocytes from splenocytes. Spleen cells were prepared from mice immunized orally with peptides (ApoB+HSP60) or KLH, after establishment of lesion, and fed a normal chow diet following tolerance induction. Representative FACS dot plots showing CD4+, CD4+ Foxp3+ and CD4+ CTLA4+ from spleen are presented (n = 6 per group). B. Plasma concentrations of TGF-β (n = 6). *P = 0.02. (TIF) [file pone.0058364.s004.tif]

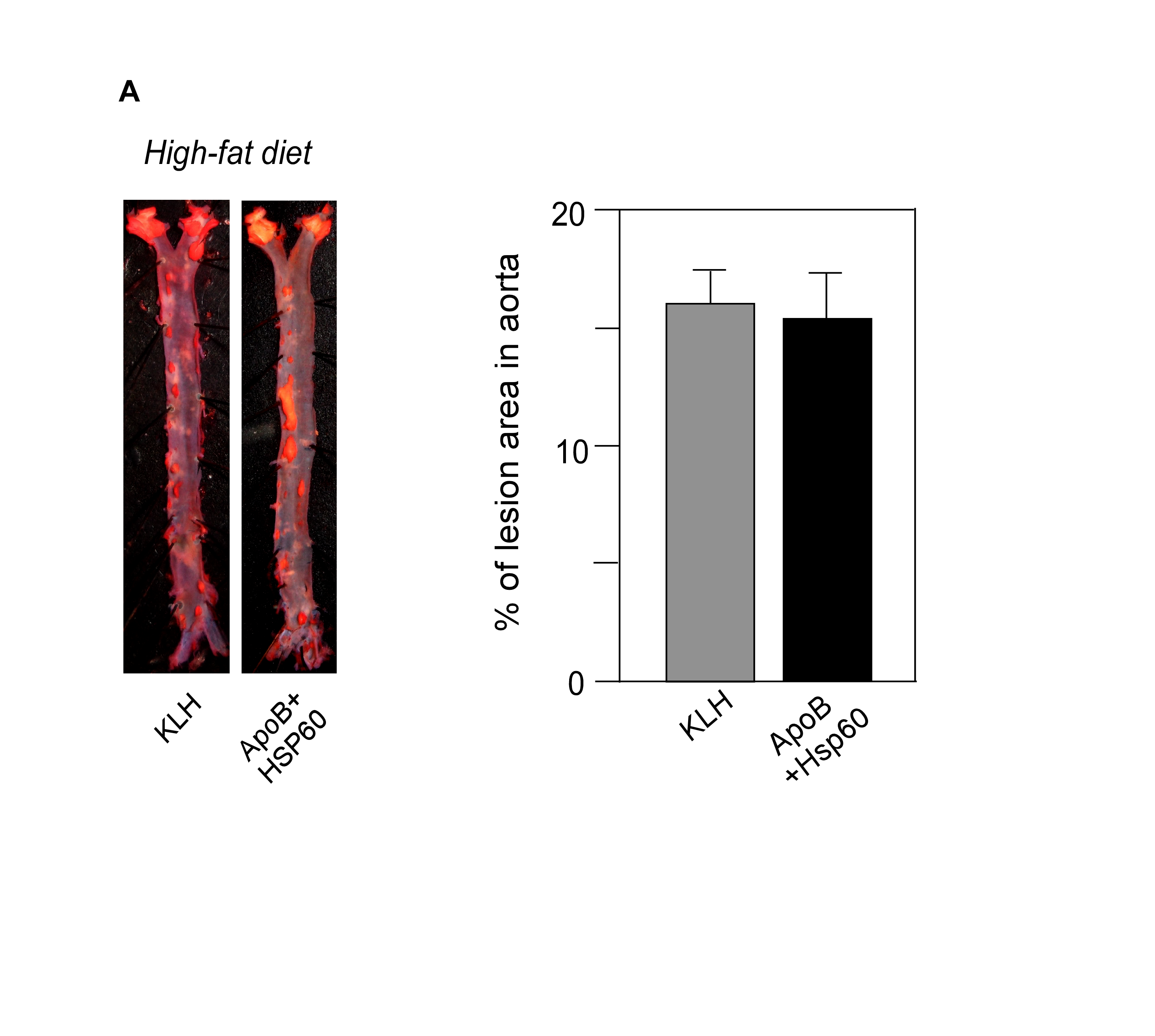

Supplement: Figure S5 — Quantification of atherosclerosis in descending aorta in mice tolerized to ApoB+HSP60 peptides with continued hyperlipidaemia. En-face analysis of the aorta from mice immunized orally with peptides (ApoB+HSP60) or KLH, after establishment of lesion, and continued on a high-fat diet following tolerance induction. Left panel. Whole aortas were collected in NBF and used for en-face analysis using Oil red-O staining. Right panel. Lesion area of the aorta was quantified relative to its surface area using Image Pro Plus software (n = 4 per group). Left panels: Representative photomicrographs of brachiocephalic plaque area stained with EVG (hearts were sectioned at the end of the study). Scale bar represents 200 µm. Right panel: Percentage of plaque area in total brachiocephalic artery, *P = 0.001 for A+H tolerized. (TIF) [file pone.0058364.s005.tif]

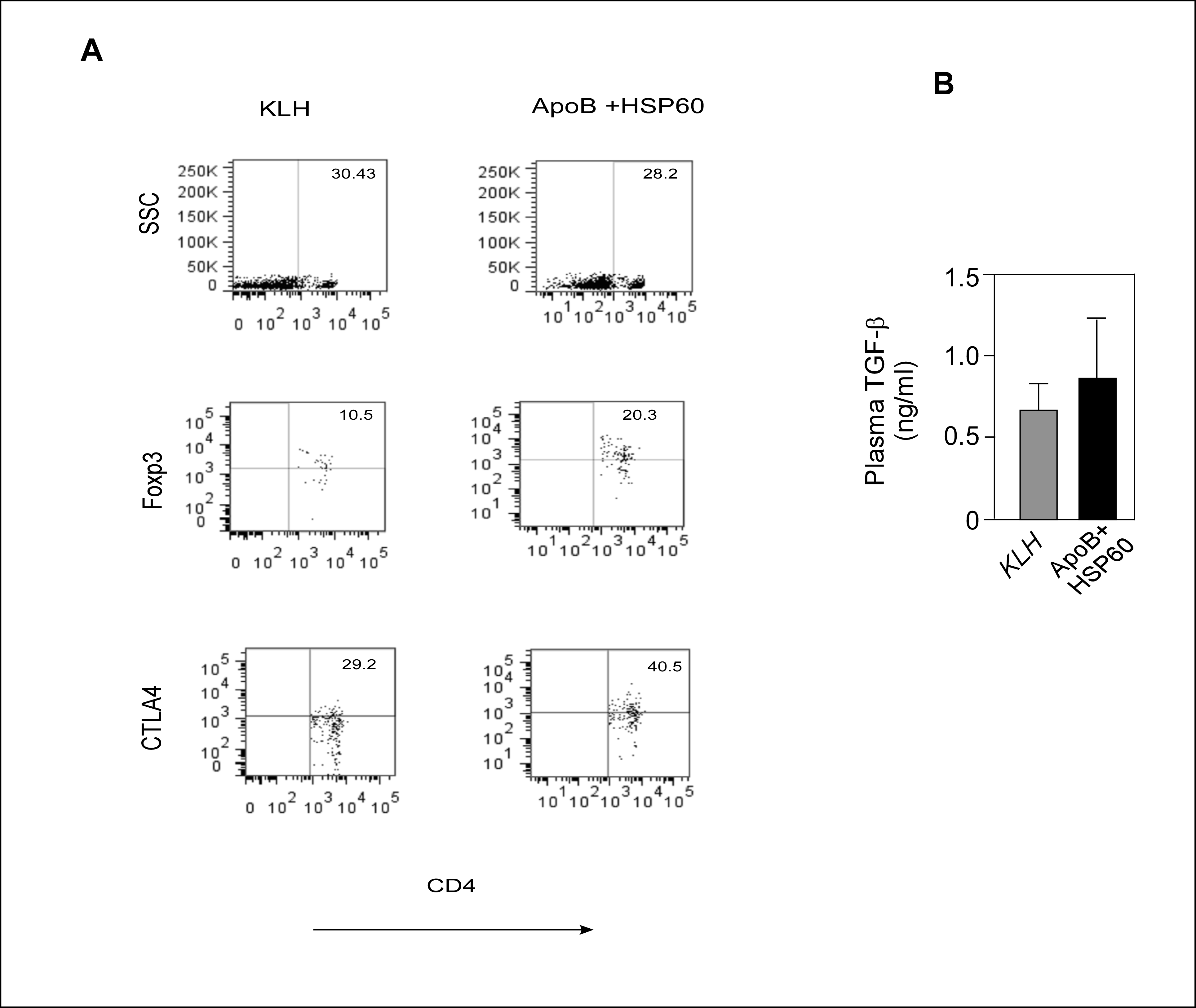

Supplement: Figure S6 — Flow cytometry analysis of splenocytes and plasma cytokine concentrations following oral tolerance to peptides with continued hyperlipidaemia. A. Flow cytometry analysis of lymphocytes from splenocytes was prepared from mice immunized orally with peptides (ApoB+HSP60) or KLH, after establishment of lesion, and continued on a high-fat diet following tolerance induction. Representative FACS dot plots showing CD4+, CD4+ Foxp3+ and CD4+ CTLA4+ from spleen cells are presented (n = 6 per group). B. Plasma concentrations of TGF-β (n = 6). P = NS. (TIF) [file pone.0058364.s006.tif]

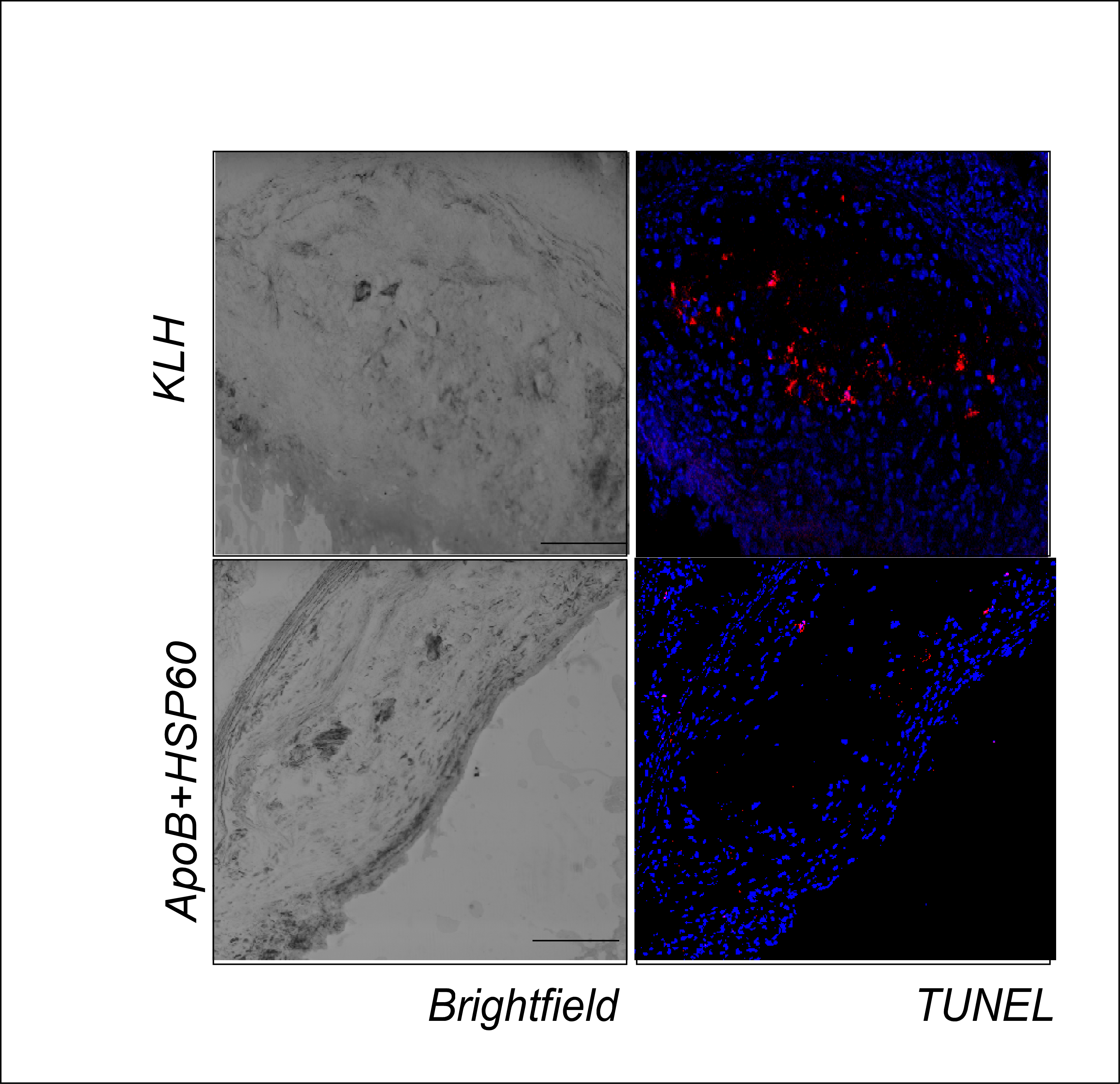

Supplement: Figure S7 — TUNEL Assay. Apoptosis as studied by TUNEL assay in aorta from mice immunized orally with peptides (ApoB+HSP60) or KLH, after establishment of lesion, and continued on a high-fat diet following tolerance induction. Aortic sections stained with TMR red (Roche Applied Science) as described by the manufacturers instructions and imaged using confocal microscope. Scale bars represent 50 µm. (TIF) [file pone.0058364.s007.tif]
