# Supplementary material for: Mucosal Tolerance to a Combination of ApoB and HSP60 Peptides Controls Plaque Progression and Stabilizes Vulnerable Plaque in Apobtm2SgyLdlrtm1Her/J Mice
Source: PLoS One. 2013 Mar 11;8(3):e58364. doi: 10.1371/journal.pone.0058364 (PMC3594317; doi:10.1371/journal.pone.0058364)
Supplement: Table S1 — Plasma lipid levels. (DOCX) [file pone.0058364.s008.docx]

# Results

## **Table S1. Plasma lipid levels**

1. Lipid levels in mice immunized with peptides and KLH, followed by 10 weeks on a high-fat diet

|  |  | **10 weeks of high-fat diet** | | | | | | |
| --- | --- | --- | --- | --- | --- | --- | --- | --- |
| **Lipids (mMol/L)** | **Basal concentration** | **KLH** | **ApoB** | | | **HSP60** | **ApoB+HSP60** | |
| Total cholesterol | 7.12±0.23 | 19.06±0.90 | | 19.36±0.35 | 18.55±0.75 | | | 17.83±0.83 |
| Triglyceride | 1.96±0.16 | 2.84±0.53 | | 2.02±0.41 | 4.20±0.42 | | | 2.77±0.55 |
| HDL | 3.16±0.15 | 4.88±0.11 | | 4.68±0.06 | 4.61±0.07 | | | 4.27±0.02 |
| LDL | 3.04±0.13 | 14.16±0.35 | | 14.85±0.18 | 12.02±0.72 | | | 11.9±1.04 |
| VLDL | 0.38±0.03 | 0.56±0.10 | | 0.40±0.08 | 0.84±0.18 | | | 0.51±0.11 |

**B.** Lipid levels in mice fed a high-fat diet for 8 weeks, tolerized, and either changed to a chow diet or continued on a high-fat diet

|  | **8 weeks of high-fat diet + 10 days of oral dosing** | **Moved to chow diet for 10 weeks** | | **Continued on a high-fat diet for 10 weeks** | |
| --- | --- | --- | --- | --- | --- |
| **Lipids (mMol/dL)** | **Baseline** | **KLH** | **A+H** | **KLH** | **A+H** |
| Total cholesterol | 14.10±0.52 | 10.79±2.65 | 9.91±0.15 | 20.98±1.67 | 21.16±1.59 |
| Triglyceride | 1.95±0.18 | 3.08±0.44 | 2.73±0.24 | 4.74±0.27 | 5.85±0.46 |
| HDL | 4.68±0.15 | 4.98±0.33 | 3.85±0.014 | 6.08±1.01 | 6.16±1.02 |
| LDL | 8.49±0.28 | 6.74±2.24 | 4.76±0.26 | 12.18±1.02 | 12.28±1.42 |
| VLDL | 0.36±0.02 | 0.70±0.06 | 0.61±0.03 | 1.17±0.28 | 1.17±0.09 |

Plasma lipid concentrations were determined using the Cobas Fara II Clinical Chemistry auto analyzer. A: Basal concentrations were determined in 5–6-week-old mice before the start of immunization. The levels were determined after the completion of study in KLH- and peptide-treated mice. B: Mice were fed a high-fat diet for 8 weeks, dosed with peptides/KLH for 10 days, and shifted to either a chow diet or continued on a high-fat diet. Lipid levels were measured before the start of oral dosing and at the end of experiment.
