# Supplementary material for: Mucosal Tolerance to a Combination of ApoB and HSP60 Peptides Controls Plaque Progression and Stabilizes Vulnerable Plaque in Apobtm2SgyLdlrtm1Her/J Mice
Source: PLoS One. 2013 Mar 11;8(3):e58364. doi: 10.1371/journal.pone.0058364 (PMC3594317; doi:10.1371/journal.pone.0058364)
Supplement: Table S2 — Antibody response to peptides. (DOCX) [file pone.0058364.s009.docx]

**Table S2. Antibody response to peptides**

|  | **Coating antigen ApoB peptide** | |
| --- | --- | --- |
|  | **IgG** | **IgA** |
| KLH | 0.033±0.002 | 0.010±0.001 |
| ApoB+ HSP60 | 0.036±0.002 | 0.013±0.009 |
|  | **Coating antigen HSP60 peptide** | |
| KLH | 0.032±0.004 | 0.013±0.008 |
| ApoB+ HSP60 | 0.039±0.002 | 0.011±0.007 |

Groups of mice were treated with a combination of peptides or KLH by the oral route. Serum antibody levels were measured 1 week and 10 weeks after the last oral dose by ELISA. Mean absorbance values ± SEM of six individual mice are given, at an antibody dilution of 1:100.

P =Not Significant
