# Supplementary material for: Mucosal Tolerance to a Combination of ApoB and HSP60 Peptides Controls Plaque Progression and Stabilizes Vulnerable Plaque in Apobtm2SgyLdlrtm1Her/J Mice
Source: PLoS One. 2013 Mar 11;8(3):e58364. doi: 10.1371/journal.pone.0058364 (PMC3594317; doi:10.1371/journal.pone.0058364)
Supplement: Method S1 — Terminal deoxynucleotidyl transferase dUTP nick end labeling (TUNEL). (DOC) [file pone.0058364.s010.doc]

# Method S1

## Terminal deoxynucleotidyl transferase dUTP nick end labeling (TUNEL)

Aortic sinus sections were permeabilized with 0.2% tritonX100 and fixed with ice-cold acetone. Sections were incubated for 1 h with freshly made solution of enzyme and dye, provided in the In Situ Cell Death Detection Kit, TMRs red (Roche Applied Science). Sections were washed with PBS, mounted with Vector shield, and imaged using a Leica DMI 4000 B confocal microscope.
