# Supplementary material for: Mucosal Tolerance to a Combination of ApoB and HSP60 Peptides Controls Plaque Progression and Stabilizes Vulnerable Plaque in Apobtm2SgyLdlrtm1Her/J Mice
Source: PLoS One. 2013 Mar 11;8(3):e58364. doi: 10.1371/journal.pone.0058364 (PMC3594317; doi:10.1371/journal.pone.0058364)
Supplement: Method S2 — Antibody response measurement. (DOC) [file pone.0058364.s011.doc]

# Method S2

## Antibody response measurement

Blood samples were collected in heparinized capillaries by retro-orbital bleeding 2 and 10 weeks after the first oral dose for assessment of antibody production. Free ApoB-100 and human HSP-60 peptides containing an N-terminal cysteine were synthesized by Severn Biotech (Worcester, UK) and were coated on Maleimide-activated 96-well plates (Pierce, Thermo Fisher Scientific Inc., USA). Peptide-specific immunoglobulin (Ig)G or IgA was measured in the serum of immunized mice using horseradish peroxidase-conjugated α-mouse IgG and horseradish peroxidase-conjugated α-mouse IgA (Sigma chemicals, St. Louis, USA) as secondary antibodies.
