# Supplementary material for: Mucosal Tolerance to a Combination of ApoB and HSP60 Peptides Controls Plaque Progression and Stabilizes Vulnerable Plaque in Apobtm2SgyLdlrtm1Her/J Mice
Source: PLoS One. 2013 Mar 11;8(3):e58364. doi: 10.1371/journal.pone.0058364 (PMC3594317; doi:10.1371/journal.pone.0058364)
Supplement: Method S3 — Real-time reverse transcription polymerase chain reaction (RT-PCR) analysis. (DOC) [file pone.0058364.s012.doc]

# Method S3

## Real-time reverse transcription polymerase chain reaction (RT-PCR) analysis

Total RNA was extracted from the ascending part of the aorta. Using microdissection scissors, fat around the ascending aorta was removed. Aortic arch was carefully microdissected and collected in RNA latter. RNA was extracted using TRIzol reagent (Invitrogen, Carlsbad, California, USA). The quality of the RNA was determined by measuring the absorbance at 230, 260, and 280 nm using Nanodrop 1000. RT-PCR was performed with two-step EXPRESS SYBR superscript RT-PCR kit (Invitrogen) using the ABI PRISM 7500 sequence detection system (Applied Biosystems, 7500 real time PCR system) according to the manufacturers protocol using the standard cycling program. Amplification reactions were performed in triplicates from RNA isolated from three mice per experimental group and the fluorescent curves were analyzed with the included software. The following mouse-specific primers were used to amplify the desired genes:

CTLA4-F GCTTCCTAGATTACCCCTTCTGC

CTLA4-R CGGGCATGGTTCTGGATCA

FOPX3-F CCCATCCCCAGGAGTCTTG

FOXP3-R ACCATGACTAGGGGCACTGTA

TGFB1-F TTGCTTCAGCTCCACAGAGA

TGFB1-R TGGTTGTAGAGGGCAAGGAC

IFNG-F ATGAACGCTACACACTGCATC

IFNG-R CCATCCTTTTGCCAGTTCCTC

CASP3- F TGTCATCTCGCTCTGGTAGC

CASP3-R AAATGACCCCTTCATCACCA

GAPDH-F AACTTTGGCATTGTGGAAGG

GAPDH-R ACACATTGGGGGTAGGAACA

MERTK-F GTGGCAGTGAAGACCATGAAGTTG

MERTK-R GAACTCCGGGATAGGGAGTCAT
